# A genome-wide map of aberrantly expressed chromosomal islands in colorectal cancer

**DOI:** 10.1186/1476-4598-5-37

**Published:** 2006-09-18

**Authors:** Eike Staub, Jörn Gröne, Detlev Mennerich, Stefan Röpcke, Irina Klamann, Bernd Hinzmann, Esmeralda Castanos-Velez, Benno Mann, Christian Pilarsky, Thomas Brümmendorf, Birgit Weber, Heinz-Johannes Buhr, André Rosenthal

**Affiliations:** 1 Max Planck Institute for Molecular Genetics, Dept. of Computational Molecular Biology., Berlin, Germany; 2 Dept. of General, Vascular and Thoracic Surgery, Charité – Campus Benjamin Franklin, Berlin, Germany; 3 Signature Diagnostics AG, Potsdam, Germany; 4 HELIOS Hospital Emil von Behring, Institute of Pathology, Berlin, Germany; 5 Boehringer Ingelheim Pharma GmbH & Co. KG, Biberach, Germany; 6 Dept. of Visceral, Thoracic, and Vascular Surgery, University Hospital Carl Gustav Carus Dresden, Germany; 7 Department of Surgery, Augusta-Kranken-Anstalt GmbH, Bochum, Germany; 8 metaGen Pharmaceuticals i.L., Berlin, Germany; 9 Present address: Novartis Institutes for BioMedical Research, Novartis Pharma AG, Basel, Switzerland; 10 Present address: immatics biotechnologies GmbH, Tübingen, Germany; 11 Present address: ALTANA Pharma AG, Preclinical Research Bioinformatics, Konstanz, Germany

## Abstract

**Background:**

Cancer development is accompanied by genetic phenomena like deletion and amplification of chromosome parts or alterations of chromatin structure. It is expected that these mechanisms have a strong effect on regional gene expression.

**Results:**

We investigated genome-wide gene expression in colorectal carcinoma (CRC) and normal epithelial tissues from 25 patients using oligonucleotide arrays. This allowed us to identify 81 distinct chromosomal islands with aberrant gene expression. Of these, 38 islands show a gain in expression and 43 a loss of expression. In total, 7.892 genes (25.3% of all human genes) are located in aberrantly expressed islands. Many chromosomal regions that are linked to hereditary colorectal cancer show deregulated expression. Also, many known tumor genes localize to chromosomal islands of misregulated expression in CRC.

**Conclusion:**

An extensive comparison with published CGH data suggests that chromosomal regions known for frequent deletions in colon cancer tend to show reduced expression. In contrast, regions that are often amplified in colorectal tumors exhibit heterogeneous expression patterns: even show a decrease of mRNA expression. Because for several islands of deregulated expression chromosomal aberrations have never been observed, we speculate that additional mechanisms (like abnormal states of regional chromatin) also have a substantial impact on the formation of co-expression islands in colorectal carcinoma.

## Background

DNA microarrays have become a standard tool for the analysis of mRNA expression levels in colorectal cancer cells. Most studies focus on the identification of differentially expressed genes in tissues at different tumor stages or on the identification of new tumor subclasses and their diagnostic gene expression signatures [1-6]. In contrast, much less is known about the influence of chromosomal neighborhood on gene expression in tumors.

In tumors different genetic mechanisms are known to affect gene expression in wider chromosomal regions. Chromosomal aberrations, like homozygous and heterozygous deletions or amplifications, alter the DNA copy number of large genomic regions or even whole chromosome arms, leading to inactivation of tumor suppressor genes [7,8] or to activation of oncogenes. Another genetic phenomenon that is assumed to have drastic effects on gene expression in cancer cells is the aberrant alteration of chromatin structure. Methylation of genomic DNA, histone acetylation, and histone methylation are assumed to have a large impact on the accessibility of DNA for transcription initiation [9]. Such epigenetic mechanisms can affect large genomic regions by possibly either silencing or activating large arrays of genes. However, the regulatory mechanisms governing chromatin assembly and disassembly are only beginning to emerge. So far, due to methodological limitations it has not been possible to study the role of such phenomena for gene expression in cancer cells on a genome-wide scale. Nevertheless, evidence from single-gene focused studies suggests that chromatin regulation does play an important role in tumorigenesis [10,11].

Regardless of which mechanism leads to coordinated expression in chromosomal domains, solely the knowledge about such domains is of considerable importance. Such knowledge could guide further studies that aim to differentiate between those differentially expressed genes that cause tumorigenesis and are the primary targets of regional genomic aberrations and those that are rather the outcome than the cause of tumor development. The rationale for the existence of such piggy-back genes is the following. The silencing of genes at close distance to a known tumor suppressor gene (TSGs) would in many cases just be a side effect of TSG silencing. A similar reasoning applies to oncogenes that can be activated by increased expression: genes that are co-amplified could also be expressed at higher levels although they do not contribute to tumorigenesis. Typical searches for differentially expressed genes by microarrays usually ignore such piggy-back effects. This may lead to the identification of large numbers of differentially expressed genes (DEGs), of which only a smaller fraction is causative for tumor development.

Though some experimental data recently became available linking microarray expression with DNA copy number analyses in some solid tumors [12-16] the knowledge about the existence of genomic islands of coordinated expression in colorectal carcinoma (CRC) is still limited. During the preparation of this manuscript a first assessment of chromosomal expression patterns in CRC in conjunction with genome-wide DNA copy number analyses became available [17]. Tsafrir et al. described a correlation of gene copy number and expression for both, deleted and amplified genes. They claimed that the described alterations become more frequent as the tumors progress from benign to metastatic forms, highlighting the need for a more precise characterization of regions of coordinate expression and gene copy number change. In addition to this most recent work, a substantial body of literature on chromosomal aberrations in CRC has accumulated [7,15,18-25] that could help to interpret findings on islands of coordinated chromosomal expression.

The need for a more precise definition of chromosomal regions of altered gene expression prompted us to find a new approach to investigate chromosomal co-expression domains in CRC. The focus of our study was the identification of up- or down-regulated gene expression in primary colon carcinoma cells compared to normal colon epithelia of the same patient. By using laser capture microdissection (LCM) we aimed to investigate transcript abundance in relatively pure cell populations, trying to minimize the influence of contaminating stroma tissue or infiltrating peripheral blood cells on expression measurements. The use of Affymetrix DNA microarray technology allowed us to simultaneously assess mRNA levels of all known human genes using only small amounts of cells obtained by LCM. Finally, we developed a new bioinformatic approach to identify regions of chromosomal deregulation which enabled the most precise survey of chromosomal expression domains in colon cancer available today. In particular, we were interested in the question whether our data correlated with the data of Tsafrir et al. who performed genome scale arrayCGH and chip-based expression analyses on a different set of colorectal cancer patients [17]. In contrast to Tsafrir et al. we put more emphasis on the identification of precise boundaries of expression domains and therefore we consider our work as complementary to their pioneering study.

## Results

### Evaluation of data set quality by tissue-wise hierarchical clustering

**Figure 1:**
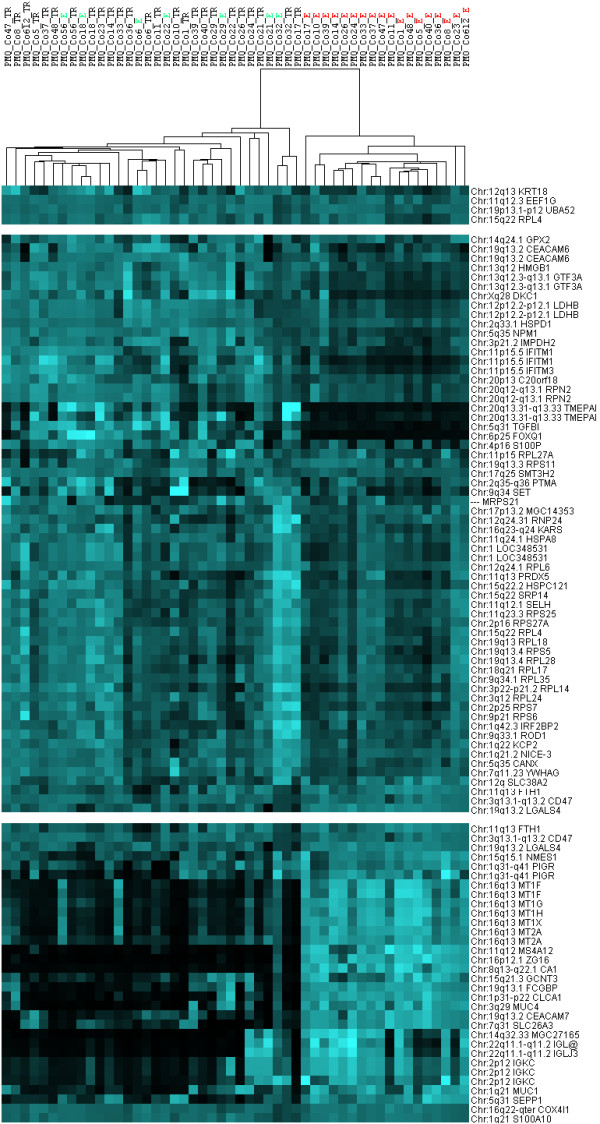
**Hierarchical clustering of samples from colorectal tumors and normal colon epithelia**. On the right, you find the chromosomal localization of the genes and the official HUGO symbol or prospective Affymetrix cluster ID. On the top, the binary tree of tissue samples based on gene expression is given. The tissue denominators either contain TR for tumor or E for epithelium and a code reflecting the identity of each patient. In the center, the expression values after normalization have been color-coded: light blue means high expression, black means low (or no) expression. Note that only a representative fraction of the 514 genes is visualized here (white bars replace some portions of original heat map). The right cluster contains only samples from normal colon epithelia, the left cluster is composed primarily of tumors along with some interspersed normal epithelial samples. Note that misplaced normal tissue (E) samples often cluster along with matching tumor (TR) samples from the same patient.

Prior to the analysis of chromosomal expression domains, we aimed to check whether the quality of our complete array expression data set (> 44 k genes) allows to extract discrepancies between tumor samples and normal epithelial tissues. Purely unsupervised hierarchical clustering of tissue samples based on gene expression vectors can provide such information. The use of the full set of 44 k genes for clustering is not desirable, because of high signal-noise ratios and computational considerations. Therefore, we pre-selected potentially informative genes for hierarchical clustering. We selected only genes which had reliable information about genomic localization and for which probe sets exceeded a minimum expression threshold in at least 20% of the experiments. To enrich informative genes for tissue distinction, we required a minimum standard deviation across all 50 samples. The pre-selection resulted in 514 probe sets. Note that we avoided to pre-select genes based on differential expression between tumor and normal tissue. We applied three rounds of normalization to genes and arrays. Finally, we applied standard centroid hierarchical clustering (Pearson correlation) to this dataset. Two large clusters were revealed (Figure 1). 18 out of 25 normal tissues formed one single cluster. The remaining 8 normal tissues mainly clustered together with matching tumor samples from same patients. This suggests that coalescence between tumor and normal samples from the same patients could be due to patient-specific gene expression characteristics. As the majority of normal samples could be clearly separated from tumors, we concluded that our data set is well suited to explore differences in gene expression between normal and tumor cells of colorectal origin.

### Global search for chromosomal islands with up- or down-regulation

**Figure 2:**
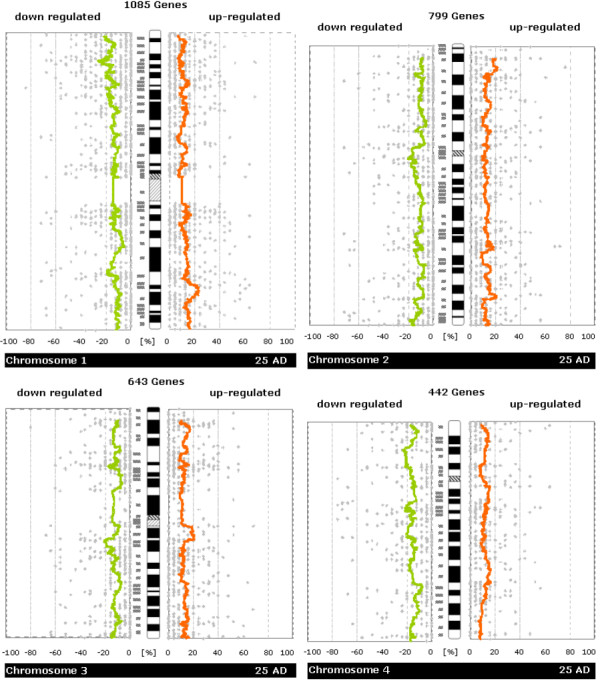
**Whole-chromosome plots of running average of fractions of samples showing up-/down-regulation in tumor versus normal samples (Chromosomes 1, 2, 3, 4)**. For each chromosome you see a separate figure. Gray dots denote the number of patients with up- or down-regulation for a single gene. Orange/green lines represent a running average of these values. The plots are made to be easily comparable with whole-genome CGH plots (like e.g. those in Knösel et al. [21]) Further details of plot construction are described in the methods section.

**Figure 3:**
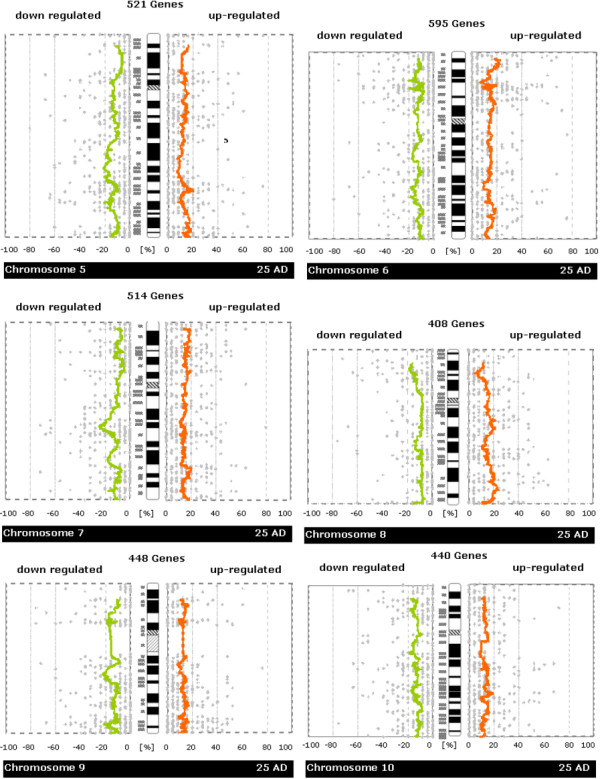
**Whole-chromosome plots of running average of fractions of samples showing up-/down-regulation in tumor versus normal samples (Chromosomes 5, 6, 7, 8, 9, 10)**. For each chromosome you see a separate figure. Gray dots denote the number of patients with up- or down-regulation for a single gene. Orange/green lines represent a running average of these values. The plots are made to be easily comparable with whole-genome CGH plots (like e.g. those in Knösel et al. [21]) Further details of plot construction are described in the methods section.

**Figure 4:**
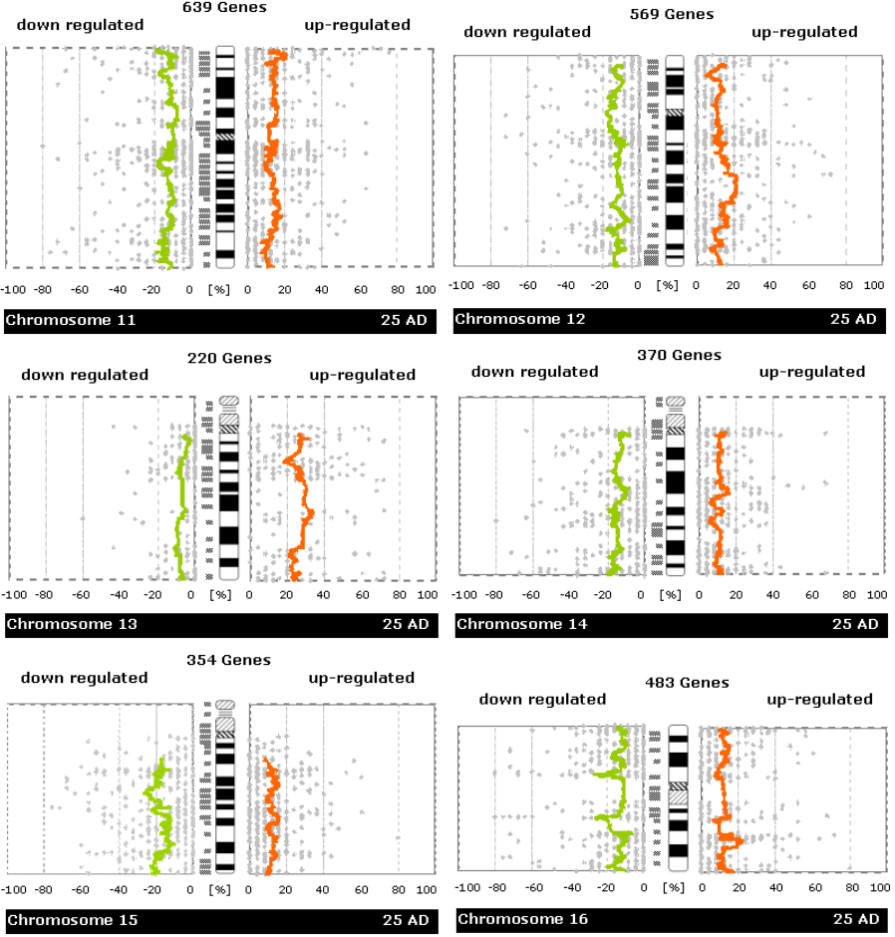
**Whole-chromosome plots of running average of fractions of samples showing up-/down-regulation in tumor versus normal samples (Chromosomes 11, 12, 13, 14, 15, 16)**. For each chromosome you see a separate figure. Gray dots denote the number of patients with up- or down-regulation for a single gene. Orange/green lines represent a running average of these values. The plots are made to be easily comparable with whole-genome CGH plots (like e.g. those in Knösel et al. [21]) Further details of plot construction are described in the methods section.

**Figure 5:**
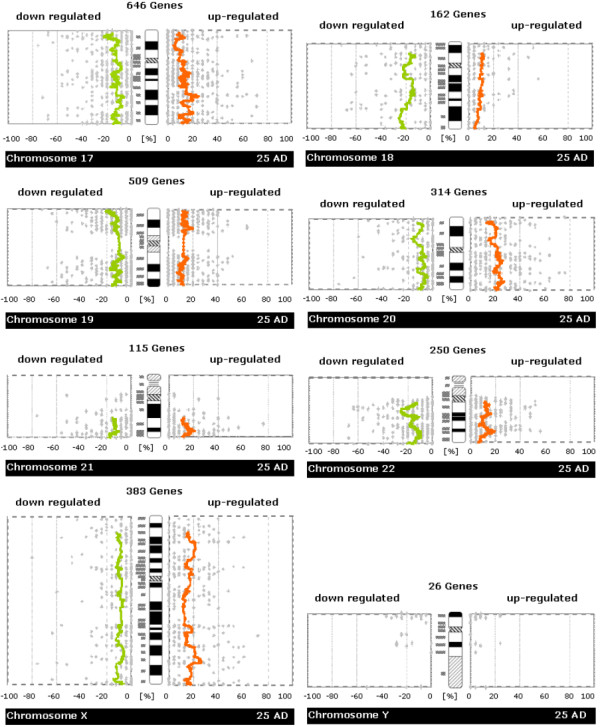
**Whole-chromosome plots of running average of fractions of samples showing up-/down-regulation in tumor versus normal samples (Chromosome 17,18,19,20,21,22,X,Y)**. For each chromosome you see a separate figure. Gray dots denote the number of patients with up- or down-regulation for a single gene. Orange/green lines represent a running average of these values. The plots are made to be easily comparable with whole-genome CGH plots (like e.g. those in Knösel et al. [21]) Further details of plot construction are described in the methods section.

Chromosome-scale analysis of gene expression (see Figures 2, 3, 4, 5) already suggested that there are many regions of misregulated expression in our CRC samples. The detailed analysis of expression along the chromosome in windows of sizes 5, 11, 21, 31, 41, 51 genes resulted in the identification of 251 partially overlapping intervals of up- or down-regulation (see Additional file 1). These intervals were condensed in 81 non-overlapping regions of expression imbalance: 43 regions with loss of expression and 38 regions with gain of expression (see Table 1). We determined the fraction of affected genes on each chromosome (see Table 2). In total, 25.3% of all genes under consideration show expression imbalance. Slightly more genes lie in chromosomal regions that show loss of expression (13.3%) than gain (12%) of expression. The fractions of genes with gain or loss of expression vary strikingly from chromosome to chromosome. Chromosomes 9, 10, 15, 18, and 22 showed only regional expression loss, whereas 8, 13, 20, and X showed only regional increase in expression. There were too few informative genes on chromosome Y to carry out a full analysis using all window sizes, but small window sizes did not reveal significant deregulation.

**Table 1:** Individual chromosomal islands of up- or down-regulation.

***expression change***	***start region***	***end region***	***start gene***	***end gene***	***potential tumor genes, hereditary CRC, known chromosomal imbalances***
loss	1p36.33	1p36.32	TNFRSF18	ARHGEF16	amplification of 1p36.33-p32 in CRC [25] // deletion of 13p36.3 in 25% of neuroblastomas and 87% of cell lines [45] // loss of expression and genomic deletion on 1p [17]
loss	1p36.13	1p36.11	PADI1	DKFZP434L0117	E2F2 // ID3 // loss of 1p36.1 in CRC [22, 25] // loss of expression and genomic deletion on 1p [17]
loss	1p35.1	1p34.3	HDAC1	PSMB2	LCK at 1p35.1 // hereditary CRC at 1p35 (OMIM 114500) // loss of expression and genomic deletion on 1p [17]
**gain**	**1q32.1**	**1q41**	**PIGR**	**DKFZp547M236**	**1q32 amplification involving MDM4 and CNTN2 in malignant gliomas [46]**
**gain**	**2p25.3**	**2p24.2**	**Hs.8379.0**	**VSNL1**	**hereditary CRC at 2p25 (OMIM 114500)**
loss	2p11.2	2q12.1	MAT2A	MGC11332	83% loss of 2p11 in mantle cell lymphoma [47] // loss in mantle cell lymphoma [48]
**gain**	**2q31.3**	**2q32.2**	**SSFA2**	**NAB1**	**hereditary HNPCC3 at 22q31-q33 (OMIM 600258) // familial breast cancer at 2q (OMIM 114480)**
**gain**	**2q33.2**	**2q35**	**Hs.163603**	**AAMP**	**familial breast cancer at 2q (OMIM 114480)**
loss	2q37.3	2q37.3	SCLY	FARP2	familial breast cancer at 2q (OMIM 114480)
**gain**	**3p25.3**	**3p25.1**	**KIAA0121**	**CAPN7**	**amplification of 3p25.2 in CRC [25] // RAF1 at 3p25.2 // FBLN1 at 3p25.2**
**gain**	**3p25.1**	**3p24.2**	**RAFTLIN**	**THRB**	
**gain**	**3p24.2**	**3p23**	**FLJ20604**	**CLASP2**	
loss	3p21.31	3p21.31	CELSR3	NPR2L	hereditary HNPCC2 at 3p21.3 (OMIM 609310) // RASFF1
**gain**	**3p11.1**	**3q13.11**	**MGC26717**	**ALCAM**	**frequent 3q11.2-q13.1 amplifications in cervix carcinomas [49]**
loss	3q13.13	3q21.2	Hs.23762.0	ITGB5	
loss	4p15.32	4p14	LAP3	Hs.118993	deletions of 4p14 in CRC [24] // SLIT2 at 4p15 is inactivated by hypermethylation in gliomas [33] // SLIT2 suppresses tumor growth [32] // loss of expression and genomic deletion on 1p [17]
loss	4q13.2	4q13.3	YT521	CXCL6	global loss of expression and genomic deletion on 4 [17]
loss	4q21.21	4q22.3	PRKG2	LIM	transition of follicular B cell lymphoma to diffuse large cell lymphoma accompanied by 4q21-q23 deletions // global loss of expression and genomic deletion on 4 [17]
loss	4q34.1	4q35.2	HPGD	Hs.130535	deletion of 4q34-q35 in colorectal cancer cell lines [25] // CASP3 at 4q34.3 // global loss of expression and genomic deletion on 4 [17]
loss	5q15	5q23.2	Hs.444378	Hs.97104	hereditary colorectal adenoma and carcinoma 1 (CRAC1) (OMIM 601228) at 15q15.3-q221 // APC at 5q21 // loss of expression and genomic deletion on 5q [17]
**gain**	**5q31.1**	**5q31.3**	**HTGN29**	**Hs.443121**	**loss of expression and genomic deletion on 5q [17]**
loss	5q31.3	5q33.1	NDFIP1	FLJ10290	amplification of 5q32-q34 in prostate cancer [50] // PDGFRB at 5q32 // loss of expression and genomic deletion on 5q [17]
**gain**	**5q33.2**	**5q35.1**	**MRPL22**	**FBXW1B**	**amplification of 5q32-q34 in prostate cancer [50] // loss of expression and genomic deletion on 5q [17]**
**gain**	**6p25.3**	**6p24.2**	**DUSP22**	**NEDD9**	**amplification of 6p25 in 24% of mantle cell lymphomas [47] // amplification of 6p25 in 75% of prostate cancers [51]**
loss	6p22.3	6p22.2	CAP2	SLC17A4	most frequent amplification of 6p22.3 in bladder cancer arrayCGH study [52]
loss	6p21.32	6p21.32	PBX2	RAB2L	
**gain**	**6p21.31**	**6p21.2**	**HMGA1**	**RNF8**	**CDKN1A at 6p21.2 // PIM1 at 6p21.2**
**gain**	**6q23.3**	**6q24.2**	**DUFD1**	**Hs.12565**	**amplification of 6q23-q24 assosicated with short survival [22]**
**gain**	**7p22.3**	**7p21.3**	**FLJ23471**	**ICA1**	**hereditary HNPCC4 at 7p22 (gene PMS2) // gain of expression and genomic amplification of 7 [17]**
**gain**	**7p21.2**	**7p15.3**	**ETV1**	**OSBPL3**	**amplification of 7p21 in mantle cell lymphomas [47] // amplification of 7p21 in osteosarcoma [53] // gain of expression and genomic amplification of 7 [17]**
**gain**	**7p14.3**	**7p13**	**LSM5**	**NPC1L1**	**gain of expression and genomic amplification of 7 [17]**
loss	7q11.23	7q21.3	SRCRB4D	CAS1	amplification of 7q11.1-q12 in metastatic CRC [24] // gain of expression and genomic amplification of 7 [17]
**gain**	**7q31.31**	**7q33**	**FAM3C**	**MGC5242**	**prostate cancer aggressiveness linked to 7q32-q33 [54] // gain of expression and genomic amplification of 7 [17]**
**gain**	**8q11.23**	**8q21.11**	**ATP6V1H**	**ANKTM1**	**amplifications of 8q11-q24 in metastasing CRC [29] // LYN at 8q12.1 // MOS at 8q12.1 // familial breast cancer at 8q11 (OMIM 114480) // amplifications at 8q in CRC [18, 21, 23, 25] // gain of expression and genomic amplification of 8q [17]**
**gain**	**8q22.3**	**8q24.22**	**TIEG**	**SLA**	**amplifications of 8q11-q24 in metastasing CRC [29] // MYC at 8q24.21 // PVT1 at 8q24.21 // amplification of 8q23-q24 in prostate cancer [55]// gain of expression and genomic amplification of 8q [17]**
loss	9p21.3	9p21.1	IFNA4	SMU-1	loss of 9p21 in CRC [25] // TUBE1 at 9p21 // CDKN2A alias p16INK4A at ??? // frequent deletion of 9p21 in prostate cancer [56] // deletion of 9p21.3 in bladder cancer [57]
loss	9p13.3	9p13.3	BAG1	OPRS1	frequent LOH at 9p13-p21 in melanoma [58]
loss	9q21.11	9q21.32	Hs.173519.0	Hs.522256	loss of 9q21-q22 in mantle cell lymphoma [59]
loss	9q34.11	9q34.11	FLJ14596	GPR107	ABL1 protooncogene at 9q34.12
loss	10p15.3	10p12.2	Hs.255096	Hs.57079.0	frequent LOH of 10p15 in gastric cancer [60] // telomerase repressor at 10q15.1 [61] // deletion of 10p14 in mantle cell lymphoma [47, 59] // OPTN at 10p14 [62]
loss	10q11.21	10q11.23	Hs.173866.0	MOB	RET at 10q11.21 // LOH in prostate cancer at 10q11.21 [51]
loss	11p15.5	11p15.5	RNH	MUC5AC	hereditary CRC at 11p15.5 / HRAS at 11p15.5 // 11p15.5 methylation-dependent expression silencing and imprinting in phaeochromocytomas [63]
**gain**	**11p15.5**	**11p15.4**	**CTSD**	**SSA1**	**CTSD (Cathepsin D) at 11p15.5 // familial breast cancer at 11p15.5 (OMIM 114480)**
**gain**	**11p13**	**11p12**	**Hs.120054.0**	**TRAF6**	**WT1 at 11p13**
**gain**	**11p11.2**	**11q12.1**	**ch-TOG**	**CTNND1**	
loss	11q13.2	11q13.4	LOC338692	SKD3	BCL1 at 11q13.3 (anti-apoptotic, amplified in breast cancer) // CCND1 at 11q13.3 (amplified in breast cancer [64]) // FGF3 at 11q13
**gain**	**11q14.1**	**11q21**	**Hs.26339.0**	**MTMR2**	
loss	11q23.3	11q23.3	AMICA	HYOU1	frequent loss of 11q23.3-q25 in neuroblastoma [65] // loss of 11q23 in 33% of 73 tumor types [66]
loss	12p13.31	12p13.2	TPI1	CLEC1	CDKN1B (alias p27Kip1) at 12p13.2
loss	12p12.3	12q12	CGI-26	MADP-1	familial breast cancer at 12p12.1 (OMIM 114480)
**gain**	**12q14.2**	**12q22**	**Hs.132260.0**	**Hs.403150**	**MDM2 at 12q15 // validated up-regulation of GPR49 at 12q21.1**
**gain**	**12q22**	**12q23.3**	**USP44**	**KIAA1033**	
loss	12q23.3	12q24.11	SART3	Hs.18370.0	loss of 12q24 in pancreas tumors [55]
**gain**	**13q14.11**	**13q22.1**	**LOC283508**	**PIBF1**	**RB1 at 13q14.2 // ARLT1 at 13q14 // gain of expression and genomic amplification of 13q [17]**
**gain**	**14q22.1**	**14q22.2**	**PSMC6**	**AND-1**	**14q22-q23 losses in 25% of tumor types [66]**
loss	14q24.1	14q24.3	Hs.369329	Hs.169812	hereditary CRC at 14q24.3 (OMIM 114500) // loss of 14q24-31 in CRC metastases [36] // FOS at 14q24.3 // hereditary HNPCC7 at 14q24.3 (gene MLH3) (OMIM) // poor prognosis when 14q24-q31 is lost in renal cell carcinoma [67] // loss of expression and genomic DNA of 14q [17]
loss	14q32.33	14q32.33	ZFYVE21	Hs.248015.0	14q32 is a tumor suppressive region in esophagal cancer [68] // loss of expression and genomic DNA of 14q [17]
loss	15q21.1	15q22.31	FBN1	CLPX	association between loss of 15q21.1-q22.2 and survival in hepatocellular carcinoma [69] // allelic imbalance at 15q21.1 in breast cancer metastases [70] // loss of expression and genomic DNA of 15q [17]
loss	15q26.1	15q26.3	GABARAPL3	FLJ25222	loss of expression and genomic DNA of 15q [17]
loss	16p12.1	16p11.2	GTF3C1	PRSS8	
loss	16q12.1	16q13	TRF4-2	FLJ13154	
**gain**	**16q22.1**	**16q22.2**	**PSMB10**	**KIAA0931**	**CDH1 (E-Cadherin) at 16q22.1**
loss	17p13.3	17p13.2	RPA1	DHX33	loss of 17p13.2 in CRC [25] // DHX33 at 17p13.2
loss	17p13.1	17p11.2	GAS7	COPS3	Near TP53 at 17p13.1 .// hereditary CRC at 17p11.2 (OMIM 114500) // hereditary CRC at 17p13.1 (OMIM 114500) // // familial breast cancer at 17p13 (OMIM 114480) // loss of 17p12 in CRC [25] // ELAC2 at 17p11.2
**gain**	**17q21.33**	**17q23.2**	**TOB1**	**PPM1D**	**NME1 (NME23) at 17q21.33 // familial breast cancer at 17q22-q23 (OMIM 114480) //**
loss	18p11.21	18q12.1	MGC24180	DSC3	loss of expression and genomic DNA of 18 [17]
loss	18q21.1	18q23	CGBP	MBP	DCC at 18q21.3 (OMIM 120470) // loss of 18q21.1 in CRC cell lines [25] // SMAD2 // SMAD4 mutations in CRC [71] // loss of expression and genomic DNA of 18 [17]
loss	19p13.3	19p13.3	DF	APCL	
**gain**	**19p13.2**	**19p13.12**	**FLJ20244**	**NOTCH3**	
**gain**	**19p13.11**	**19q13.12**	**LOC114977**	**TYROBP**	**NIFIE14 at 19q13.12**
loss	19q13.2	19q13.32	MGC20255	TOMM40	AKT2 (breast carcinoma at 19q13.2 // TGFB1 at 19q13.2 // proapototic Bax at 19q13.33
**gain**	**20p11.21**	**20q11.21**	**C1QR1**	**BCL2L1**	
**gain**	**20q11.22**	**20q11.23**	**RNPC2**	**C20orf102**	**SRC at 20q11.23 (overexpressed in breast carcinoma) // gain of expression and genomic DNA of 20q [17]**
**gain**	**20q13.12**	**20q13.33**	**SLC12A5**	**ARFRP1**	**gain of expression and genomic DNA of 20q [17]**
**gain**	**21q22.12**	**21q22.3**	**C21orf18**	**TMPRSS2**	**ETS2 at 21q22.2**
loss	21q22.3	21q22.3	PFKL	COL6A1	COL18A1 (Endostatin) at 21q22.3
loss	22q11.21	22q12.1	SDF2L1	TPST2	familial breast cancer at 22q12.1 (OMIM 114480)
loss	22q13.31	22q13.33	Hs.296370.0	RABL2B	hereditary CRC at 22q13 (OMIM 114500)
**gain**	**Xp22.13**	**Xp22.11**	**SCML1**	**ARX**	
**gain**	**Xp11.22**	**Xp11.1**	**Hs.3383.1**	**Hs.224455**	
**gain**	**Xq24**	**Xq26.3**	**FLJ32122**	**CXX1**	

These are condensed results of the ChARM analyses: overlapping regions with evidence for up- or down-regulation from various analyses of different cross-correlation window sizes have been fused into single regions. The original ChARM output including p values for each region and additional annotation can be found in Additional file 1. Hereditary colorectal cancer syndromes are indicated along with their OMIM ID. Gene symbols are official or provisional HUGO symbols if available, otherwise names of Unigene clusters. Information about known tumor genes in misregulated regions were extracted from the literature. Tumor-associated genes are located within expression islands or in near vicinity.

**Table 2:** Statistics on expression imbalances across human chromosomes.

***Chromosome***	***Genes Considered***	***Genes In Regions With Expression Imbalance***	***Genes In Regions With Expression Gain***	***Genes In Regions With Expression Loss***
chr1	2976	418 (14.0%)	109 (3.7%)	309 (10.4%)
chr2	2236	550 (24.6%)	296 (13.2%)	254 (11.4%)
chr3	1815	444 (24.5%)	203 (11.2%)	241 (13.3%)
chr4	1273	347 (27.3%)	0 (0.0%)	347 (27.3%)
chr5	1496	489 (32.7%)	219 (14.6%)	270 (18.0%)
chr6	1767	355 (20.1%)	240 (13.6%)	115 (6.5%)
chr7	1537	585 (38.1%)	451 (29.3%)	134 (8.7%)
chr8	1152	311 (27.0%)	311 (27.0%)	0 (0.0%)
chr9	1215	193 (15.9%)	0 (0.0%)	193 (15.9%)
chr10	1255	271 (21.6%)	0 (0.0%)	271 (21.6%)
chr11	1781	515 (28.9%)	328 (18.4%)	187 (10.5%)
chr12	1584	546 (34.5%)	279 (17.6%)	267 (16.9%)
chr13	683	180 (26.4%)	180 (26.4%)	0 (0.0%)
chr14	1061	206 (19.4%)	27 (2.5%)	179 (16.9%)
chr15	951	306 (32.2%)	0 (0.0%)	306 (32.2%)
chr16	1279	349 (27.3%)	103 (8.1%)	246 (19.2%)
chr17	1807	329 (18.2%)	120 (6.6%)	209 (11.6%)
chr18	546	246 (45.1%)	0 (0.0%)	246 (45.1%)
chr19	1636	339 (20.7%)	194 (11.9%)	145 (8.9%)
chr20	917	386 (42.1%)	386 (42.1%)	0 (0.0%)
chr21	358	118 (33.0%)	68 (19.0%)	50 (14.0%)
chr22	736	192 (26.1%)	0 (0.0%)	192 (26.1%)
chrX	1042	217 (20.8%)	217 (20.8%)	0 (0.0%)
chrY	96	0 (0.0%)	0 (0.0%)	0 (0.0%)

TOTAL	31199	7892 (25.3%)	3731 (12.0%)	4161 (13.3%)

Here, estimates of portions of chromosomes that are affected by regional regulation of expression are given. The second column gives the number of genes on a particular chromosome that were included in our analysis. The following columns contain the numbers of genes that are located in deregulated expression islands (up/down).

### Individual chromosomal islands with gain of expression

#### 8q11.23-q21.13

**Figure 6:**
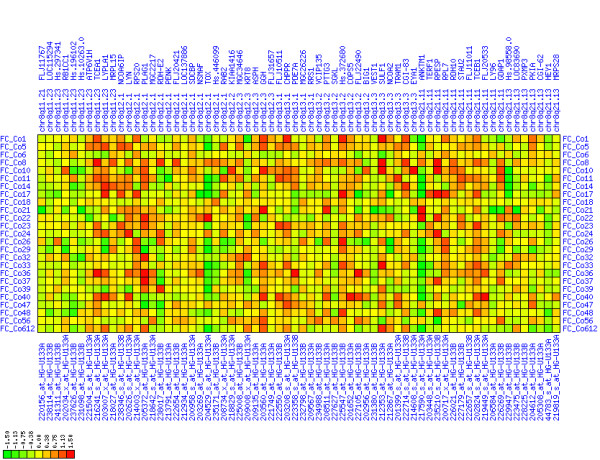
**Up-regulation of mRNA expression in human chromosomal region 8q11.23-q21.13 (T/N relative expression heat map)**. Heat map of fold change of tumor-versus-normal expression. Genes are given in chromosomal order on the horizontal axis. Patient codes are given on the vertical axis. The legend depicts which colors code for which expression changes on a log_e _scale (green: down in tumor; red: up in tumor). View in conjunction with Figures 7 and 8.

**Figure 7:**
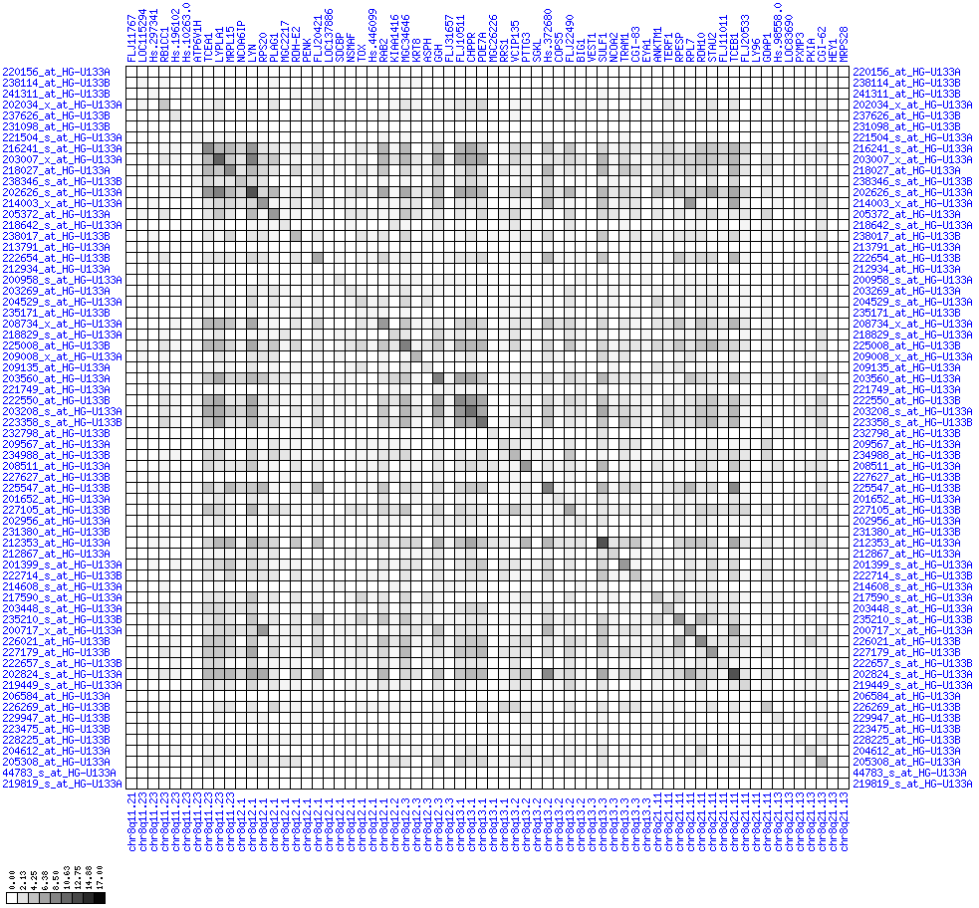
**Up-regulation of mRNA expression in human chromosomal region 8q11.23-q21.13 (patient counts with coordinate up-regulation)**. Grayscale plot of cross-comparison of up-regulation patterns across patients for gene pairs in a particular region. Both, horizontal and vertical axes comprise the same genes in chromosomal order. In each square total counts of patients with consistent up-regulation in two genes are coded by different shades of gray. Dark squared regions along the diagonal indicate coordinated regulation in patient subgroups. Note, that many more patients show up-regulation as indicated by dark spots in this figure than down-regulation as indicated by dark spots in Figure 8. The left region of exceptionally strong up-regulation spans TCEA1, LYPLA1, MRPL15, the known tumor gene LYN, and PLAG1. Note that TOX and ANKTM1 are down-regulated in approximately half of the tumor samples.

**Figure 8:**
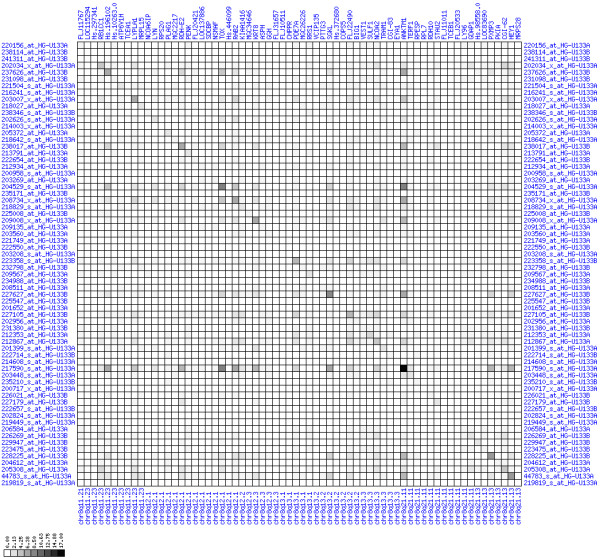
**Up-regulation of mRNA expression in human chromosomal region 8q11.23-q21.13 (patient counts with coordinate down-regulation)**. Grayscale plot of cross-comparison of down-regulation patterns across patients for gene pairs in a particular region. Both, horizontal and vertical axes comprise the same genes in chromosomal order. In each square total counts of patients with consistent down-regulation in two genes are coded by different shades of gray. Dark squared regions along the diagonal indicate coordinated regulation in patient subgroups. View in conjunction with Figures 6 and 7.

Gain of expression in region 8q11.23-q21.13 is strongest in a small interval (8q12.1) that spans genes from TCEA1 to PLAG1 (see Figures 6, 7, 8). There have been numerous reports of copy number gains of chromosome 8q in CRC [18,21,23,25] which suggests a possible mechanism leading to over-expression in our patients. The known blood cell oncogene LYN is located in this interval and it is up-regulated in several of our tumor samples. It has been reported before that LYN is expressed in colorectal tumors [26]. The concerted up-regulation of LYN along with other genes in this region suggests a role for LYN in CRC. Another interesting gene in this interval is PLAG1 (pleomorphic adenoma gene 1) for which chromosomal aberrations have been described that lead to over-expression in salivary gland tumors [27,28]. No informative expression measures were obtained for the MOS protein kinase gene which is located between RPS20 and PLAG1, although this may be due to technical limitations. Genes encoding components of the translation machinery, the mitochondrial ribosomal protein MRPL15 and cytosolic ribosomal proteins RPL7 and RPS20, are located in this region, highlighting the need for enhanced translation in cancer cells. The concomitant down-regulation of the TOX and ANKTM1 genes in many patients in an environment of transcriptional activation is remarkable, but the functional significance remains unclear. Buffart et al. have reported amplifications of 8q11-q24 in metastasizing CRC [29], highlighting a possible mechanism for gain of expression in this region. In summary, our analysis suggests that chromosomal region 8q12.1 is a candidate target region for genetic alterations that lead to over-expression in CRC.

#### 20q11.22-q11.23

**Figure 12:**
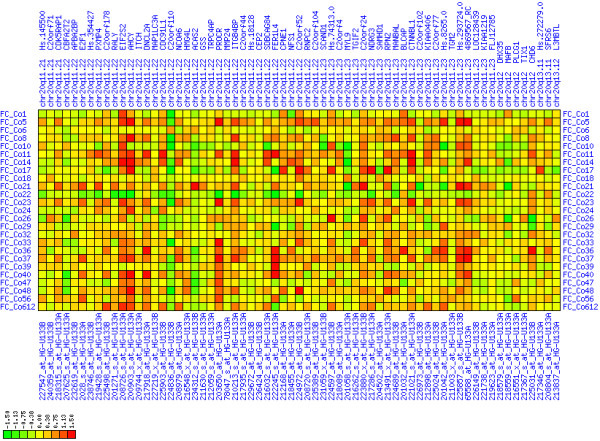
**Up-regulation of mRNA expression in human chromosomal region 20q11.22-q11.23 (T/N relative expression heat map)**. Heat map of fold change of tumor-versus-normal expression. Genes are given in chromosomal order on the horizontal axis. Patient codes are given on the vertical axis. The legend depicts which colors code for which expression changes on a log_e _scale (green: down in tumor; red: up in tumor). View in conjunction with Figures 13 and 14.

**Figure 13:**
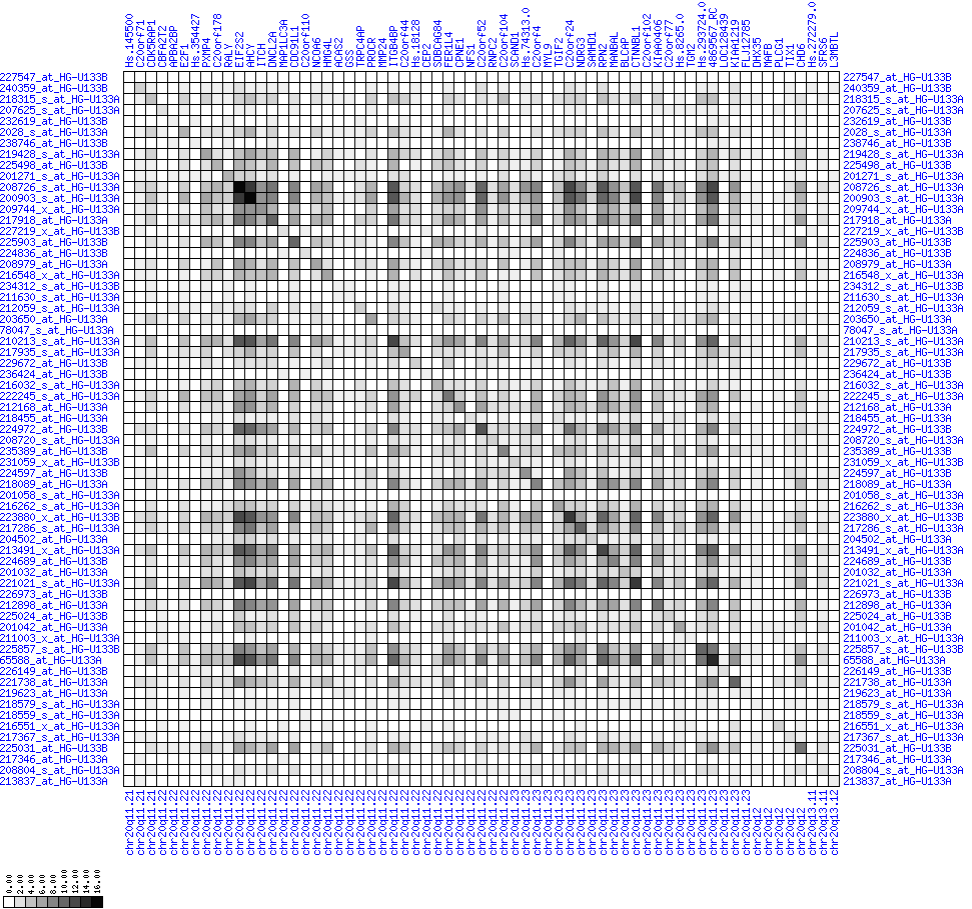
**Up-regulation of mRNA expression in human chromosomal region 20q11.22-q11.23 (patient counts with coordinate up-regulation)**. Grayscale plot of cross-comparison of up-regulation patterns across patients for gene pairs in a particular region. Both, horizontal and vertical axes comprise the same genes in chromosomal order. In each square total counts of patients with consistent up-regulation in two genes are coded by different shades of gray. Dark squared regions along the diagonal indicate coordinated regulation in patient subgroups. Note, that many more patients show up-regulation as indicated by dark spots in this plot than down-regulation as indicated by dark spots in Figure 14. The known most frequently up-regulated genes in this region are EIF2S2, AHCY, ITCH, DNCL2A, ITG4BP, C20orf24, NDRGL3, RPN2 and CTNNBL1. Also note the gene C20orf110 alias TP53INP2 which is down-regulated in the majority of tumors.

**Figure 14:**
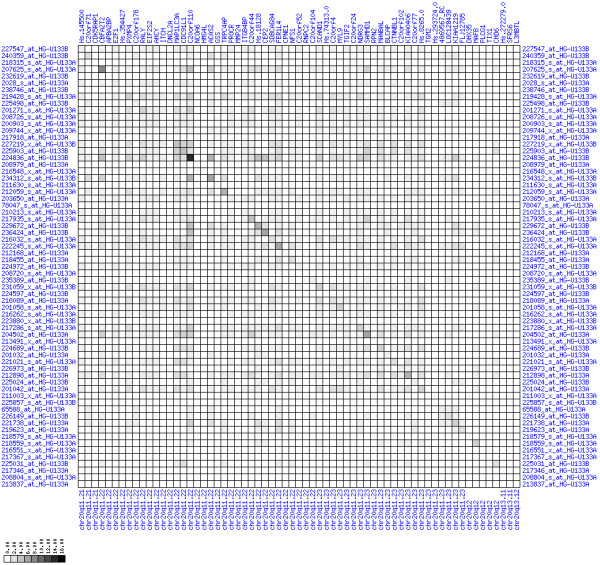
**Up-regulation of mRNA expression in human chromosomal region 20q11.22-q11.23 (patient counts with coordinate down-regulation)**. Grayscale plot of cross-comparison of down-regulation patterns across patients for gene pairs in a particular region. Both, horizontal and vertical axes comprise the same genes in chromosomal order. In each square total counts of patients with consistent down-regulation in two genes are coded by different shades of gray. Dark squared regions along the diagonal indicate coordinated regulation in patient subgroups. View in conjunction with Figures 12 and 13.

The region 20q11.22-q11.23 was among the most frequently up-regulated regions (see Figures 12, 13, 14). Amplifications of regions on chromosome 20q have been identified independently by several groups in CRCs [19,21,23,24]. The interval comprises the known tumor gene SRC (located between MANBAL and BLCAP in Figures 12, 13, 14) for which no informative expression measures were obtained. We note that it is possible that the SRC gene is the primary target of up-regulation in our CRC patients, the up-regulation of other genes being just piggy-back effects. However, also the up-regulation of the CTNN1L1 transcript could be of potential functional significance for CRC development. CTNN1L1 shows partial homology to the known colorectal cancer gene beta-catenin in the armadillo repeat region and has a nuclear localization signal, suggesting that it could play an important role in signal transduction to the nucleus in CRC. Also up-regulation of the E3 ubiquitin ligase Itchy (ITCH) could be of potential importance as selective ubiquitin-tagging of signaling proteins for destruction is an emerging mechanism in cancer biology. The need for accelerated protein synthesis in cancer cells is reflected by the up-regulation of the translation initiation factor EIF2S2. Remarkably, we found dramatic down-regulation against the regional trend of C20orf110 alias p53-inducible protein 2 (TP53INP2) whose expression is usually positively controlled by the p53 protein. For unknown reasons p53 seems to be unable to induce TP53INP2 expression in the majority of CRCs studied here.

#### 12q14.2-12q22

**Figure 15:**
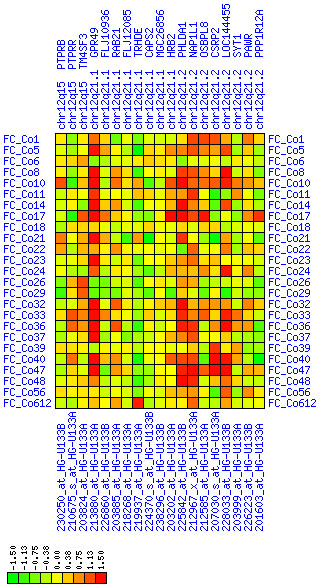
**Up-regulation of mRNA expression in human chromosomal region 12q21.1-q21.2 (T/N relative expression heat map)**. Heat map of fold change of tumor-versus-normal expression. Genes are given in chromosomal order on the horizontal axis. Patient codes are given on the vertical axis. The legend depicts which colors code for which expression changes on a log_e _scale (green: down in tumor; red: up in tumor). View in conjunction with Figures 16 and 17.

**Figure 16:**
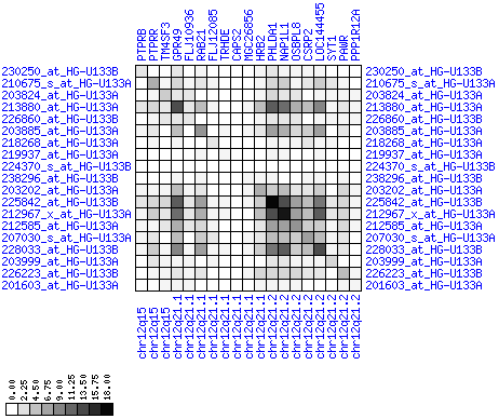
**Up-regulation of mRNA expression in human chromosomal region 12q21.1-q21.2 (patient counts with coordinate up-regulation)**. Grayscale cross-comparison plots of up-regulation patterns across patients (analogous to Figures 7, 10 and 13). View this plot in conjunction with Figures 15 and 17. Note, that many more patients show up-regulation as indicated by dark spots in this plot than down-regulation as indicated by dark spots in Figure 17. Over-expressed genes in this region comprise leucine-rich G-protein coupled receptor 5 (GPR49), HIV-1 rev binding protein 2 (HRB2), pleckstrin-homology-like domain family A member 1 (PHLDA1), nucleosome assembly protein 1-like 1 (NAP1L1), oxysterol binding protein-like 8 (OSBPL8), cystein- and glycine-rich protein 2 (CSRP2), E2F transcription factor 7 (LOC144455).

**Figure 17:**
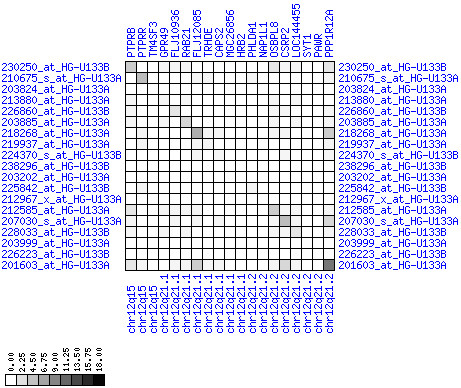
**Up-regulation of mRNA expression in human chromosomal region 12q21.1-q21.2 (patient counts with coordinate down-regulation)**. Grayscale cross-comparison plots of down-regulation patterns across patients (analogous to Figures 8, 11, 14). View this plot in conjunction with Figures 15 and 16.

We observed increased expression of genes in chromosomal region 12q14.2-12q22 (see Figures 15, 16, 17). The MDM2 gene at 12q15 is a possible target of this misregulation. However, within this large region there is a smaller region at 12q21.1-q21.2 spanning eight genes that exhibit exceptionally high expression in our tumor samples. Among these is LGR5 alias GPR49, a G-protein coupled receptor that has large leucine-rich repeats in its N-terminus. We could confirm the up-regulation of GPR49 in CRC by quantitative PCR and *in-situ *hybridization (data not shown). This finding and the exceptional suitability of G-protein-coupled receptors as drug targets make the LGR5/GPR49 protein a potential target for future therapeutical approaches. We do not know of any other reports that link this region to CRC.

#### 17q21.33-17q23.2

**Figure 18:**
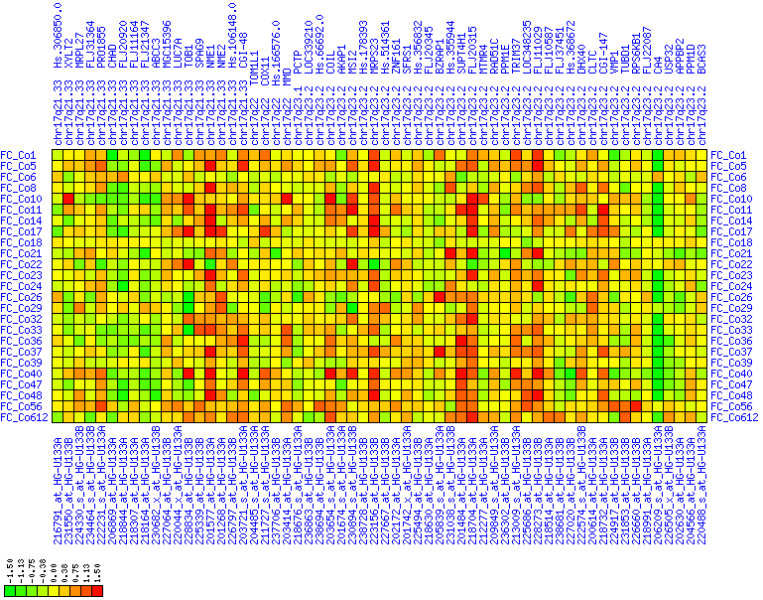
**Up-regulation of mRNA levels in human chromosomal region 17q21.33-23.2 (T/N relative expression heat map)**. Heat map of fold change of tumor-versus-normal expression. Genes are given in chromosomal order on the horizontal axis. Patient codes are given on the vertical axis. The legend depicts which colors code for which expression changes on a log_e _scale (green: down in tumor; red: up in tumor). View in conjunction with Figures 19 and 20.

**Figure 19:**
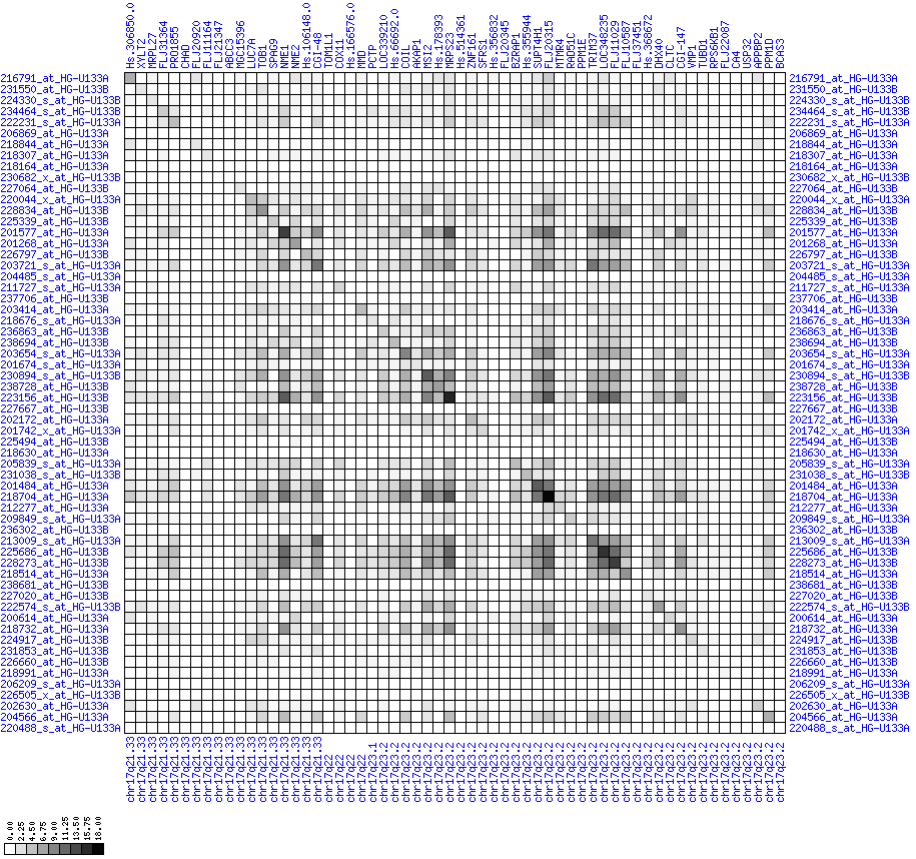
**Up-regulation of mRNA levels in human chromosomal region 17q21.33-23.2 (patient counts with coordinate up-regulation)**. Grayscale cross-comparison plots of up-regulation patterns across patients (analogous to Figures 7, 10, 13). View this plot in conjunction with Figures 18 and 20. Note, that many more patients show up-regulation as indicated by dark spots in this plot than down-regulation as indicated by dark spots in Figure 20. This region has been reported in other studies to be frequently amplified in colon cancer (see Table 3).

**Figure 20:**
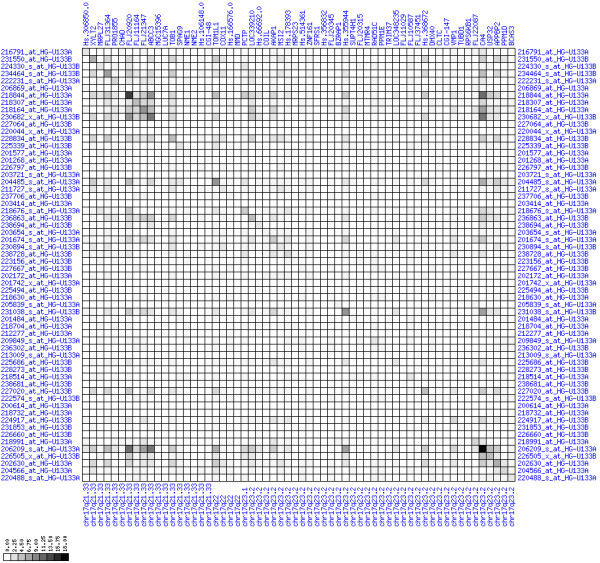
**Up-regulation of mRNA levels in human chromosomal region 17q21.33-23.2 (patient counts with coordinate down-regulation)**. Grayscale cross-comparison plots of down-regulation patterns across patients (analogous to Figures 8, 11, 14). View this plot in conjunction with Figures 18 and 19.

The chromosomal interval 17q21.33-17q23.2 harbors numerous up-regulated genes (see Figures 18, 19, 20). Chromosomal gains of this region in CRC have been described by two independent studies [21,25]. Up to 18 of 25 patients show up-regulation of expression in this region. The known tumor gene NME1 (non-metastatic 1; encoding the NM23A protein, a nucleoside diphosphate kinase) is among the most frequently up-regulated genes in this region. Also the paralogous genomic neighbor NME2 which acts in the same pathway is strongly up-regulated. These two genes are possibly the primary targets of regional expression up-regulation. However, up-regulation of several other genes is also remarkable. The up-regulation of the mitochondrial ribosomal component MRPS23 is notable as it is in agreement with other observations of up-regulation of genes acting in translation (see above). Additionally, the RING finger gene FLJ20315/RNF124, possibly encoding a novel E3 ubiquitin ligase, and the suppressor of Ty 4 homologue 1 (SUPT4H1), a putative human chromatin regulator that alters transcription, are genes that are strongly up-regulated and could have the potential to contribute to development of CRC.

### Individual chromosomal islands with loss of expression

#### 1p36.13-1p36.11

**Figure 9:**
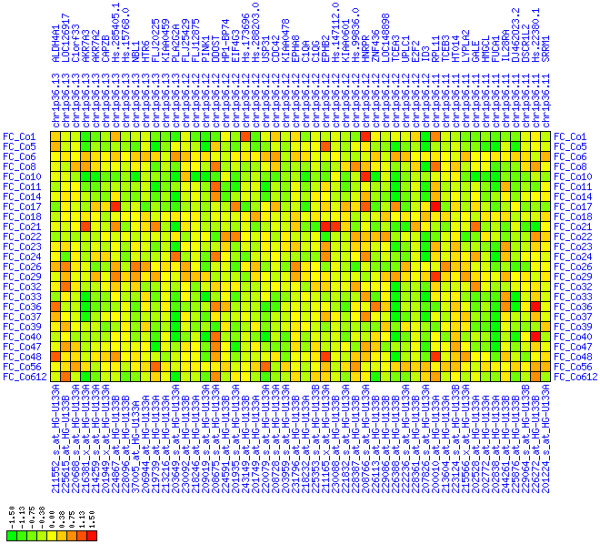
**Down-regulation of mRNA expression in human chromosomal region 1p36.13-1p36.11 (T/N relative expression heat map)**. Heat map of fold change of tumor-versus-normal expression. Genes are given in chromosomal order on the horizontal axis. Patient codes are given on the vertical axis. The legend depicts which colors code for which expression changes on a log_e _scale (green: down in tumor; red: up in tumor). View in conjunction with Figures 10 and 11.

**Figure 10:**
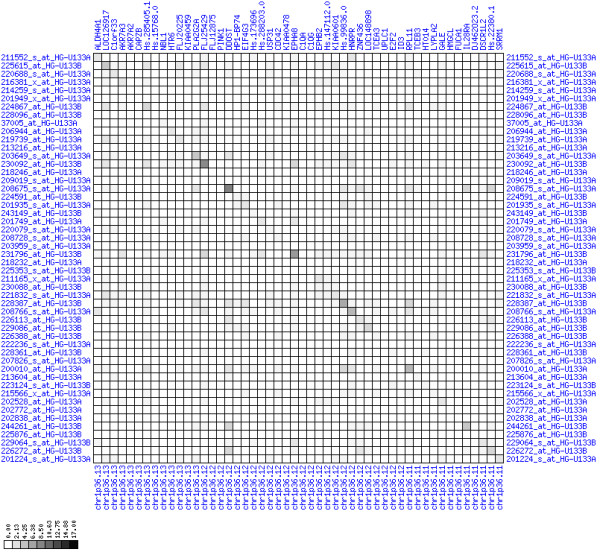
**Down-regulation of mRNA expression in human chromosomal region 1p36.13-1p36.11 (patient counts with coordinate up-regulation)**. Grayscale plot of cross-comparison of up-regulation patterns across patients for gene pairs in a particular region. Both, horizontal and vertical axes comprise the same genes in chromosomal order. In each square total counts of patients with consistent up-regulation in two genes are coded by different shades of gray. Dark squared regions along the diagonal indicate coordinated regulation in patient subgroups. View this plot in conjunction with Figures 9 and 11.

**Figure 11:**
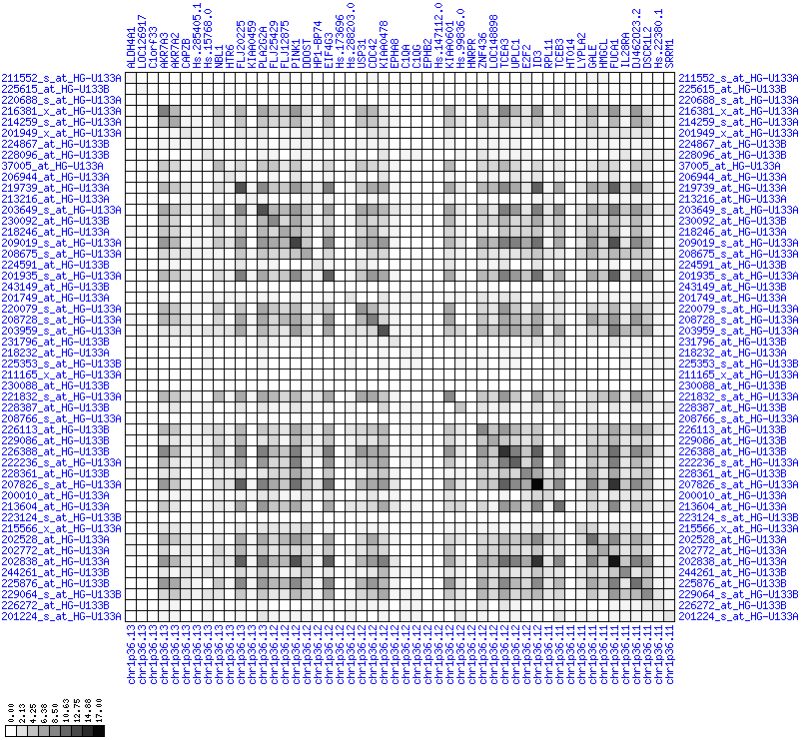
**Down-regulation of mRNA expression in human chromosomal region 1p36.13-1p36.11 (patient counts with coordinate down-regulation)**. Grayscale plot of cross-comparison of down-regulation patterns across patients for gene pairs in a particular region. Both, horizontal and vertical axes comprise the same genes in chromosomal order. In each square total counts of patients with consistent down-regulation in two genes are coded by different shades of gray. Dark squared regions along the diagonal indicate coordinated regulation in patient subgroups. Note, that many more patients show down-regulation as indicated by dark spots in this plot than up-regulation as indicated by dark spots in Figure 10. This region has been reported in other studies to be frequently deleted in colorectal cancer (see Table 4). This is the most significantly down-regulated region of our analysis. Note the expression of potential tumor genes PLA2G2A, E2F2, and CDC42.

The most strongly down-regulated region in our study is 1p36.13-1p36.11 (see Figures 9, 10, 11). A larger chromosome region comprising this fragment has recently been reported to be frequently deleted in CRC (see Tables 1 and 4). No tumor suppressor gene has been found yet. Our data suggest multiple genes that could act as class II TSGs. Several have been associated with proliferative processes or even cancer before. The PLA2G2A encodes phospholipase A2 group IIA which has been proposed as a TSG and a marker for metastasis and patient survival in gastric cancer [30]. The E2F2 transcription factor is a known regulator of TSGs and interacts specifically with the RB protein. It plays an important role in the cell cycle. The CDC42 protein is a small Rho-like GTPase. It acts in intracellular signaling and is involved in various processes like control of morphology, migration, endocytosis, and the cell cycle. Therefore, PLAG2A, E2F2 and CDC42 are the primary candidate tumor suppressors in this region.

**Table 4:** Expression in Islands frequently deleted in CRC.

***chromosomal region***	***fraction of patients with deletions***	***congruent with our expression data***	***CGH study***
1p		yes	[17]
1p36.2-1p36.1	21%	yes	[22]
1p21-1p22	72%	no	[21, 24]
**3p12**	**66%**	**no**	**[21, 24]**
4	–	yes	[15, 17, 18]
4p	42%	yes	[19]
4p14	87%	yes	[21, 24]
4q	58%	yes	[19]
4q27-4q28	96%	no	[21, 24]
4q31.3	39%	no	[22]
4q34.3	38%	yes	[25]
4q35	34%	yes	[22]
**5q**	**> 35%**	**yes**	**[17, 23]**
**5q21**	**81%**	**yes**	**[21, 24]**
6q16-6q21	72%	no	[21, 24]
**8p**	**37%**	**no**	**[17, 18, 23, 25]**
**8p24-8p21**	**66%**	**no**	**[21, 24]**
9p21	64%	yes	[21, 24]
9p22	25%	no	[22]
**10q26.2**	**22%**	**no**	**[25]**
11q13.1	30%	no	[25]
**14q**		**yes**	**[17]**
**14q13-14q21**	**64%**	**yes**	**[21, 24]**
15q		yes	[17]
**17p**	**46%**	**yes**	**[23]**
**17p13.2**	**51%**	**yes**	**[25]**
**17p12**	**32%**	**yes**	**[25]**
18		yes	[17]
18p	49%	yes	[23]
18q	60%	yes	[23]
18q	32%	yes	[18, 25]
18q	92%	yes	[19]
18q11.2	72%	no	[22]
18q12.2	59%	no	[25]
18q21-18q23	96%	yes	[21]
18q21.1	60%	yes	[25]
**21q**	**> 35%**	**yes**	**[23]**
**21q21**	**74%**	**no**	**[21, 24]**

Literature survey of chromosomal regions with evidence for deletions in colorectal cancers. We checked all regions of frequent chromosomal deletions for congruence with expression patterns. Congruence between literature CGH data and our expression data was declared on the presumption that allelic loss causes mRNA down-regulation.

#### 4p15.31-4p15.2

**Figure 21:**
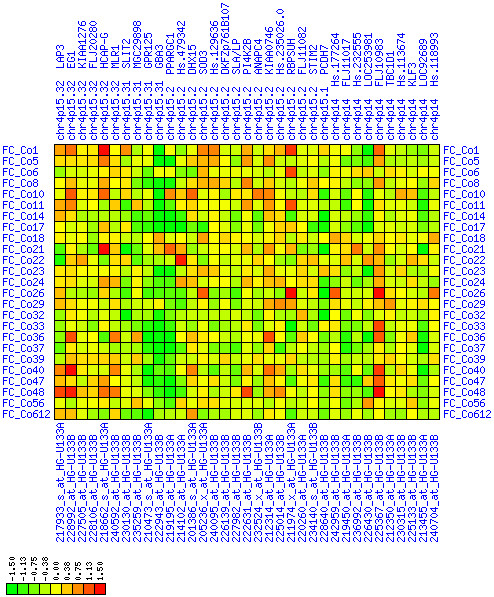
**Down-regulation of mRNA expression in human chromosomal region 4p15.31-15.2 (T/N relative expression heat map)**. Heat map of fold change of tumor-versus-normal expression. Genes are given in chromosomal order on the horizontal axis. Patient codes are given on the vertical axis. The legend depicts which colors code for which expression changes on a log_e _scale (green: down in tumor; red: up in tumor). View in conjunction with Figures 22 and 23.

**Figure 22:**
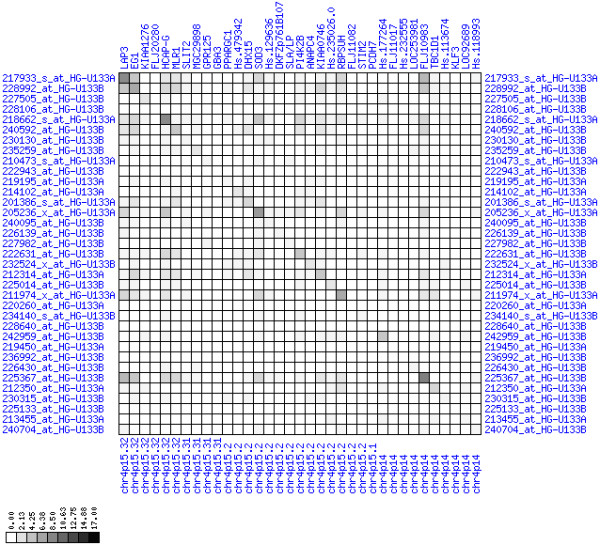
**Down-regulation of mRNA expression in human chromosomal region 4p15.31-15.2 (patient counts with coordinate up-regulation)**. Grayscale cross-comparison plot of up-regulation patterns across patients (analogous to Figures 7, 10, 13). View this plot in conjunction with Figures 21 and 23.

**Figure 23:**
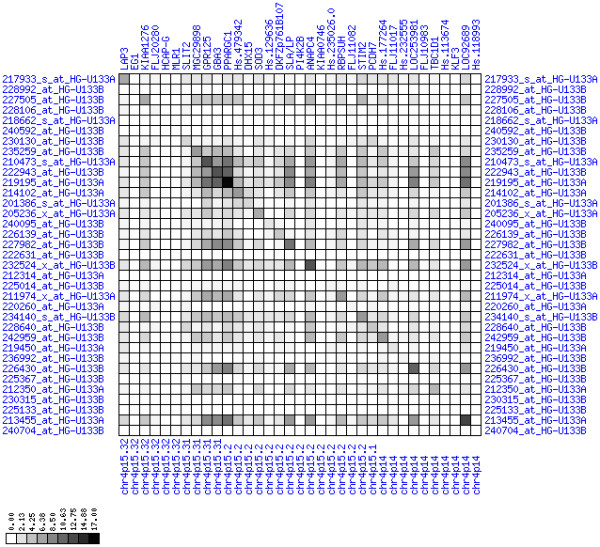
**Down-regulation of mRNA expression in human chromosomal region 4p15.31-15.2 (patient counts with coordinate down-regulation)**. Grayscale cross-comparison plot of down-regulation patterns across patients (analogous to Figures 8, 11, 14). View this plot in conjunction with Figures 21 and 22. Note, that many more patients show down-regulation as indicated by dark spots in this plot than up-regulation as indicated by dark spots in Figure 22. This region has been reported in other studies to be frequently deleted in colon cancer (see Table 4). Note the expression down-regulation of SLIT2, GPR125 and PCDH7.

The region 4p15.31-4p15.2 is part of a larger region (see Table 1) that showed marked down-regulation of expression in our tumor samples (see Figures 21, 22, 23). Full or partial losses of chromosome 4 are well known phenomena in the development of CRC [18,19,23,24]. One of the strongly down-regulated genes in this region is the SLIT2 gene at 4p15.31 that encodes a membrane protein regulating cellular migration. It has recently been described as a new tumor suppressor gene in CRC, gliomas, lung and breast tumors and seems to be transcriptionally inactivated by epigenetic silencing [31-33]. In addition, several other genes of this region could serve as candidate class II tumor suppressor genes. The GPR125 gene encodes an orphan G-protein coupled receptor that has a large extracellular N-terminus with an immunoglobulin domain and leucine-rich repeats, similar to GPR49 described above. The PCDH7 gene belongs to the protocadherin gene family. It encodes a transmembrane protein that has seven extracellular cadherin repeats, suggesting that it is involved in cellular adhesion and adhesion-dependent intracellular signaling. The functions of genes in this region suggest that this regional expression loss influences adhesion and migration properties of cancer cells. Both, epigenetic silencing and chromosomal aberrations are potential mechanisms leading to expression loss in this region.

#### 18q21.2-18q23

**Figure 24:**
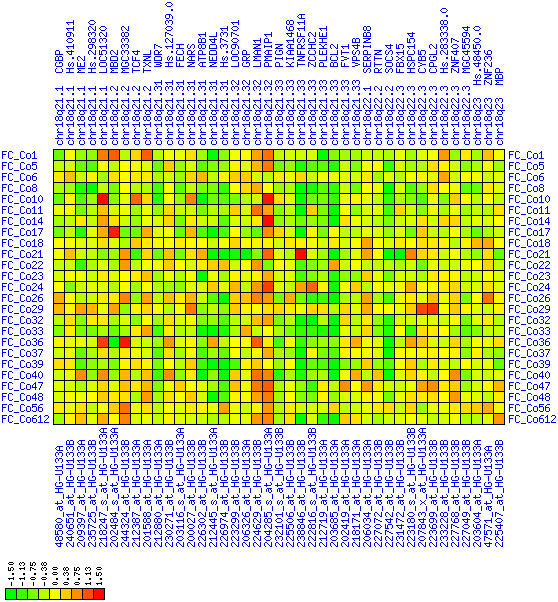
**Down-regulation of mRNA expression in human chromosomal region 18q21.2-18q23 – the BCL2 region (T/N relative expression heat map)**. Heat map of fold change of tumor-versus-normal expression. Genes are given in chromosomal order on the horizontal axis. Patient codes are given on the vertical axis. The legend depicts which colors code for which expression changes on a log_e _scale (green: down in tumor; red: up in tumor). View in conjunction with Figures 25 and 26.

**Figure 25:**
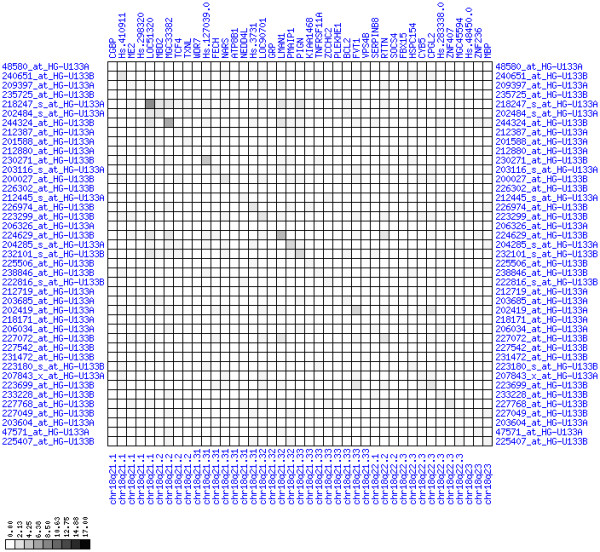
**Down-regulation of mRNA expression in human chromosomal region 18q21.2-18q23 – the BCL2 region (patient counts with coordinate up-regulation)**. Grayscale cross-comparison plot of up-regulation patterns across patients (analogous to Figures 7, 10, 13). View this plot in conjunction with Figures 24 and 26.

**Figure 26:**
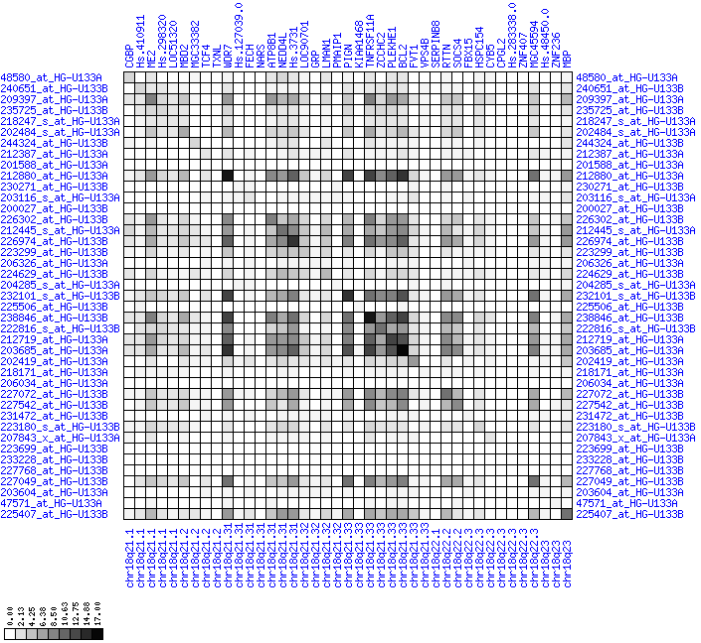
**Down-regulation of mRNA expression in human chromosomal region 18q21.2-18q23 – the BCL2 region (patient counts with coordinate down-regulation)**. Grayscale cross-comparison plot of down-regulation patterns across patients (analogous to Figures 8, 11, 14). View this plot in conjunction with Figures 24 and 25. Note, that many more patients show down-regulation as indicated by dark spots in this plot than up-regulation as indicated by dark spots in Figure 25. This region has been reported in other studies to be frequently deleted in colon cancer (see Table 4). Note the expression down-regulation of BCL2. SMAD4 (Hs.298320) and TCF4 are only weakly down-regulated. The DCC gene is also located in this region between LOC51320 and MBD2 but no informative expression measures were obtained.

There are several reports of loss of chromosome 18q in CRC (see Tables 1 and 4). We found a smaller region of expression down-regulation at 18q21.2-18q23 (see Figures 24, 25, 26). There is a hot spot for down-regulation in direct vicinity of the BCL2 gene. Its special role in cancer qualified the anti-apoptotic BCL2 protein as a therapeutic target molecule [34,35]. Here we observed down-regulation of BCL2 and its neighbors which is contradictory to its known anti-apoptotic cancer-promoting function. Distal to BCL2 at 18q21.1-18q21.2 there is a region of less pronounced down-regulation between ME2 and MBD2. The SMAD4 (Hs.298320) is only weakly down-regulated and the biological significance is questionable. The DCC (deleted in colorectal carcinoma), proximal to MBD2, is the largest gene in this region, but no statements about its expression can be made because of a lack of informative expression measures. The SMAD2 and SMAD7 genes are in close vicinity to this region. In summary, we do not have direct evidence for down-regulation of tumor suppressor genes in this region. Instead, we observed down-regulation of the cancer-promoting BCL2 gene. Therefore, the biological significance of this domain of expression loss remains elusive. Possibly, the down-regulation of the BCL2 region is just a by-stander effect of deletions targeted at DCC disruption. Alternatively, BCL2 down-regulation could be an unsuccessful attempt of the tumor cells' genetic program to shift the cellular homeostasis towards cell death.

#### 5q22.2-5q23.1

**Figure 27:**
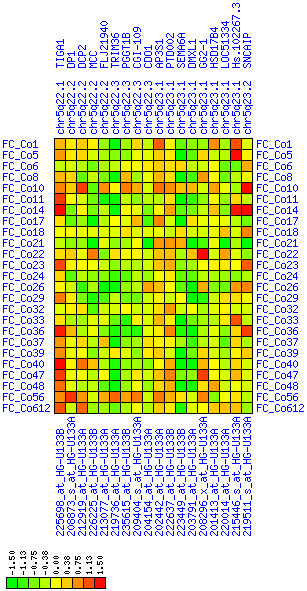
**Down-regulation of mRNA expression in human chromosomal region 5q22.2-5q23.1 – the APC region (T/N relative expression heat map)**. Heat map of fold change of tumor-versus-normal expression. Genes are given in chromosomal order on the horizontal axis. Patient codes are given on the vertical axis. The legend depicts which colors code for which expression changes on a log_e _scale (green: down in tumor; red: up in tumor). View in conjunction with Figures 28 and 29.

**Figure 28:**
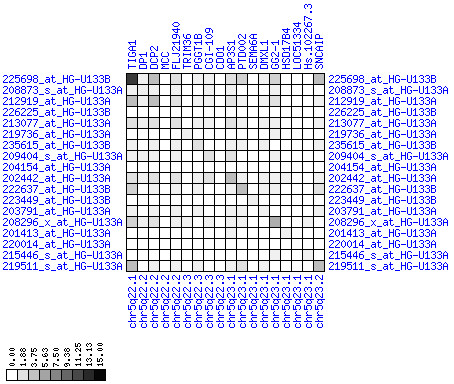
**Down-regulation of mRNA expression in human chromosomal region 5q22.2-5q23.1 – the APC region (patient counts with coordinate up-regulation)**. Grayscale cross-comparison plot of up-regulation patterns across patients (analogous to Figures 7, 10, 13). View this plot in conjunction with Figures 27 and 29.

**Figure 29:**
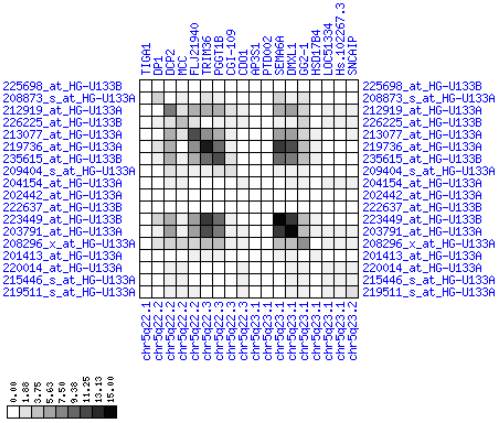
**Down-regulation of mRNA expression in human chromosomal region 5q22.2-5q23.1 – the APC region (patient counts with coordinate down-regulation)**. Grayscale cross-comparison plot of down-regulation patterns across patients (analogous to Figures 8, 11, 14). View this plot in conjunction with Figures 27 and 28. Note, that many more patients show down-regulation as indicated by dark spots in this plot than up-regulation as indicated by dark spots in Figure 28. This region has been reported in other studies to be frequently deleted in colon cancer (see Table 4). APC itself is not represented in this plot (no valid expression measures). It is located down-stream of TIGA1 and up-stream of DP1 and DCP2. Note the sharp change from expression up-regulation (TIGA1) to expression down-regulation (DCP2 to DMXL1) in this interval.

Not unexpected, we found loss of expression in region 5q22.2-5q23.1 (see Figures 27, 28, 29). This interval harbors two known TSGs in colon cancer, the adenomatous polyposis coli gene (APC) gene and the mutated in colorectal cancer (MCC). We were not able to obtain expression values for APC. APC is located at the border of a region at 5q22.2-5q22.3 that harbors several drastically down-regulated genes. Central in this region is the MCC gene. The distal border is the CDO1 gene. We assume that deletion or epigenetic silencing of this region is a frequent mechanism contributing to colorectal tumorigenesis. It is possible that also APC or MCC show reduced expression, that genes in this region other than APC and MCC are piggy-back genes, and that their misregulation is not of functional significance for tumorigenesis.

#### 14q24.3

**Figure 30:**
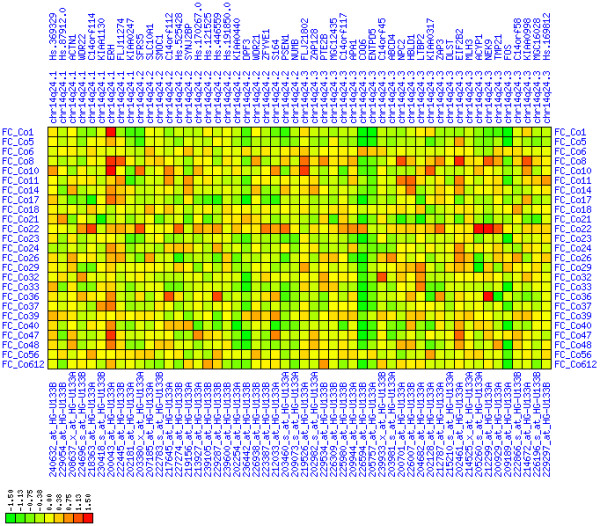
**Down-regulation of mRNA expression in human chromosomal region 14q24.1-14q24.3 – the FOS region (T/N relative expression heat map)**. Heat map of fold change of tumor-versus-normal expression. Genes are given in chromosomal order on the horizontal axis. Patient codes are given on the vertical axis. The legend depicts which colors code for which expression changes on a log_e _scale (green: down in tumor; red: up in tumor). View in conjunction with Figures 31 and 32.

**Figure 31:**
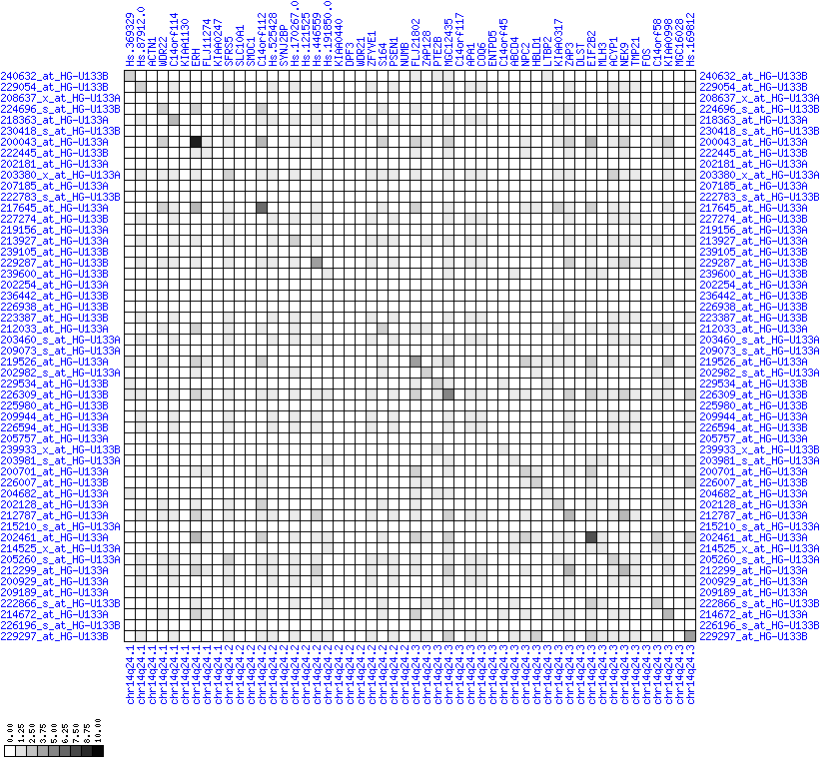
**Down-regulation of mRNA expression in human chromosomal region 14q24.1-14q24.3 – the FOS region (patient counts with coordinate up-regulation)**. Grayscale cross-comparison plot of up-regulation patterns across patients (analogous to Figures 7, 10, 13). View this plot in conjunction with Figures 30 and 32.

**Figure 32:**
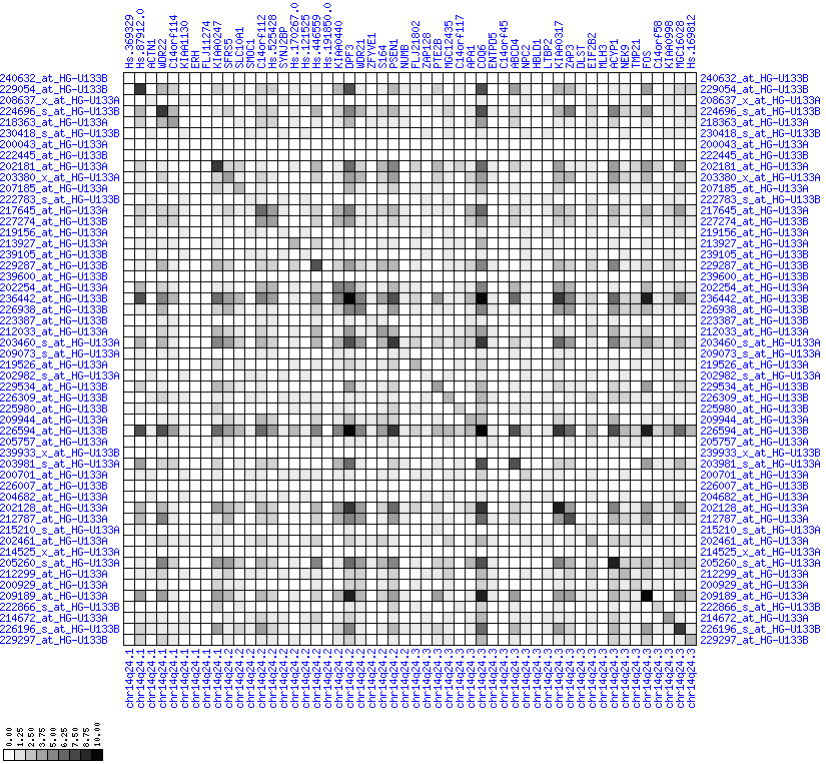
**Down-regulation of mRNA expression in human chromosomal region 14q24.1-14q24.3 – the FOS region (patient counts with coordinate down-regulation)**. Grayscale cross-comparison plot of down-regulation patterns across patients (analogous to Figures 8, 11, 14). View this plot in conjunction with Figures 30 and 31. Note, that many more patients show down-regulation as indicated by dark spots in this plot than up-regulation as indicated by dark spots in Figure 31. This region has been reported in other studies to be frequently deleted in colon cancer metastases (see Table 1). The FOS oncogene is the 5^th ^gene from the right and is one of the most strongly down-regulated genes in this region. Note the expression of MLH3, KIAA0317, KIAA0440/SIPA1L1, NUMB, SYNJ2BP and PSEN1.

The chromosomal region 14q24.3 has been implicated in colorectal cancer several times (see Table 1). We found coordinated down-regulation of expression of genes in 14q24.1-14q24.3 (see Figures 30, 31, 32). The region comprises the MLH3 gene that is linked to hereditary non-polyposis colorectal cancer type 7 (HNPCC7). We note that also the FOS gene encoding one half of the bZIP dimer activator protein (AP-1) at 14q24.3 is strongly down-regulated. FOS is known as an oncogene and its down-regulation is therefore unexpected. However, deletions of 14q24.3 have been linked to metastatic CRC [36]. In combination, these results suggest that there is a class II tumor metastasis suppressor in this region. This class II TSG is probably not MLH3, as its protein function is hardly related to cellular functions promoting metastasis. The functions of several other strongly misregulated proteins, however, make them better candidates for metastasis suppressors. KIAA0317 codes for a predicted transmembrane ubiquitin ligase. Ubiquitin ligases can help to tag misfolded transmembrane proteins in the ER for destruction via the proteasome system [37]. Absence of such a function could result in misexpressed proteins at the cell surface which could promote metastasis. Other potential candidates for metastasis suppressor genes in this region code for the transmembrane Alzheimer protein PSEN1, the GTPase activating protein KIAA0440/SIPA1L1, the PDZ-domain synaptojanin 2-binding protein SYNJ2BP and the developmental regulator and Notch interaction partner NUMB.

## Discussion

### Global analysis of chromosomal regions with expression gain or loss

We found that 25% of the genes lie in regions that are affected by expression imbalance in colon cancer. This does not mean that 25% of the genes are misregulated as many genes that fall into these regions are not expressed at all in tumors and in normal epithelium of the colon. Additionally, we note that these numbers are probably an upper limit because the sliding window approach probably included several genes in close proximity to the boundaries of misexpressed regions. Nevertheless, the number of regions of imbalanced expression is remarkable and suggest that there is extensive regulation in CRC at the genomic level. Recently, Nakao et al. estimated from genome-wide array CGH data that ~17% of the human genome is affected by DNA copy number changes in CRC [23]. Prior to a more detailed analysis of individual regions in this study, this suggested that not all regional expression changes in CRC will be explainable by DNA copy number aberrations.

There are only slightly more genes with expression loss than regions with expression gain. One can argue that a tumor ought to show a higher frequency of expression loss than expression gain. Reasons are that there should be a tendency to lose tumor suppressor genes selectively and to lose non-essential genes (genomic ballast) as a side effect. If transcription would be a process that is predominantly driven by positive regulation of transcriptional activators, one would assume that any partial genome loss results in a slow down of transcription. In the light of these considerations, an equally high number of regions with expression gain can be interpreted in two ways. Either positive selection drives expression gain of some regions in cancer cells, or a default phenotype of transcription suppression dominates in normal cells which is relaxed during tumor cell development.

### Gene expression in chromosomal regions with frequent DNA copy number changes in CRC

Most studies reported frequent gains of chromosome 7, 8q, 13q, 20q and losses of 4 and 18q in CRC [18,19,21-25]. These broadly-defined alterations are in perfect agreement with chromosome-specific trends in our expression data, especially the exclusive presence of domains of expression gain on 8, 13 and 20 and the exclusive presence of domains of expression loss on chromosome 4 and 18 (see Table 2 and Figures 21, 22, 23, 24, 25, 26). There is a single discrepancy for chromosome 7: region 7q11-7q12 has been reported as amplified in CRC, but its expression is significantly down-regulated in our tumor samples.

For a more detailed survey of congruence between gene expression and chromosomal aberrations in CRC, we compared our results to six previous studies reporting chromosomal gains or losses in distinct chromosomal regions [18,19,21-25] (see Tables 3, 4). We considered only those chromosomal regions that were reported by different researchers or were found to be aberrant in > 20% of tumor samples. In summary, we found that the majority of deletion regions show a reduction in expression. This suggests that regional transcriptional silencing in CRC is mainly achieved by loss of genomic DNA. In contrast, amplified regions rather show heterogeneous expression changes. We found regions of expression gain in the most frequently reported regions of chromosome gain on 7, 8q, 13, 20q. These regions are in support for a positive correlation of DNA copy number and transcript abundance, although a direct causal relationship is not shown in this study.

**Table 3:** Expression in Islands frequently amplified in CRC.

***chromosomal region***	***fraction of patients***	***congruence with our expression data***	***CGH study***
1		no	[18]
1q21	57%	no	[21]
**3p25.2**	**30%**	**yes**	**[25]**
**3q26.31**	**32%**	**no**	**[25]**
6p12.1	24%	no	[25]
**7**	**46%**	**yes**	**[17, 18, 25]**
**7p**	**> 35%**	**yes**	**[23]**
**7p**	**42%**	**yes**	**[19]**
**7p22.3**	**57%**	**yes**	**[25]**
**7q**	**42%**	**yes**	**[19]**
**7q**	**> 35%**	**yes**	**[23]**
**7q11.2-7q12**	**75%**	7q11.23-7q21.3	**[21, 24]**
8q		yes	[17]
8q	27%	yes	[18, 25]
8q	42%	yes	[23]
8q24	57%	yes	[21]
8q24.21	40%	yes	[25]
8q24.22	30%	yes	[25]
8q24.3	51%	yes	[25]
**9q34**	**67%**	9q31.11	**[21, 24]**
11q	> 35%	yes	[23]
**12p13.1-12p13.2**	**22%**	**down at 12p13.31-12p13-2**	**[25]**
13		yes	[18]
13	46%	yes	[25]
13q		yes	[17]
13q13	64%	no	[21, 24]
13q34	64%	no	[21]
**15q22-15q23**		**down at 15q21.1-15q22.31**	**[22]**
16p12-11	70%	down at 16p12.1-16p11.2	[21, 24]
16p13.3	49%	no	[25]
16q23.2	24%	no	[25]
**17q**	**42%**	**yes**	**[19]**
**17q12-17q21**		**yes**	**[24]**
**17q12**	**22%**	**no**	**[25]**
**17q21**	**59%**	**yes**	**[21]**
**17q21.32**	**24%**	**yes**	**[25]**
**17q25.3**	**43%**	**no**	**[25]**
19p13	70%	yes	[21, 24]
**20**		**yes**	**[18, 25]**
**20p**	**58%**	**yes**	**[19]**
**20q**		**yes**	**[17]**
**20q**	**65%**	**yes**	**[23]**
**20q**	**100%**	**yes**	**[21]**
**20q**	**92%**	**yes**	**[19]**
**20q13.3**	**56%**	**yes**	**[22]**
**20q13.3**	**81%**	**yes**	**[25]**
**20q11.2-20q13.2**		**yes**	**[24]**
22q11	61%	22q11.21-22q21.1	[21]
22q13.2	24%	no	[25]

Literature survey of chromosomal regions with evidence for amplifications in colorectal cancers. We checked all regions of frequent chromosomal amplifications for congruence with expression patterns. Congruence between literature CGH data and our expression data was declared on the presumption that allelic gain causes mRNA up-regulation.

However, there are also many regions of frequent deletions that did not show alterations in expression or that were even down-regulated (7q11.2-7q12, 9q34, 12p13.1-13.2, 15q22-15q23, 16p12-16p11, 22q11; compare Tables 3 and 4). One possible explanation is that these down-regulated regions are not amplified in our tumor samples. An alternative explanation is that the influence of chromosomal amplification on transcription levels can be either positive or negative. It is possible that amplification of a particular genomic region disrupts transcription of amplified genes by a yet unknown mechanism, e.g. by induction of chromatin-based silencing, or by separation of essential enhancer regions from transcription starts.

Platzer et al. found amplifications in 7p, 8q, 13q, 20q in 26%–43% of their CRC patients and revealed by microarray-based expression analysis that only 81 of 2146 genes in amplified regions show over-expression (3.8%) whereas 164 of 2146 genes show under-expression (7.7%). Using a different approach (microdissection, oligo arrays, analysis aimed at the identification of single chromosomal expression domains and not at the location of all differentially expressed genes in chromosomes) we found several smaller up-regulated regions and no regions of down-regulation in the same chromosomal regions. Therefore, our data partly contradicts the findings of Platzer et al. which state that in these frequently amplified regions gene expression is rather down-regulated. However, other misregulated expression domains (see above) of our study confirmed the general notion by Platzer et al. that frequently amplified regions in CRC can also exhibit down-regulation of transcript levels.

### Aberrantly expressed chromosomal islands linked to hereditary cancer

Roughly 5% of all colorectal carcinomas are hereditary non-polyposis colorectal cancers (HNPCCs). In HNPCC, histologically verified colorectal carcinoma is found in at least three relatives from two or more successive generations. In at least one patient, the age of onset should be less than 50 years. Seven chromosomal regions have been linked to HNPCC. More than half of these HNPCC regions show misregulated expression in our patients. Three regions show down-regulation (3p21.3, 2q31-q33 comprising PMS1, 14q24.3 comprising MLH3), one region shows up-regulation (7p22 comprising PMS2), and three regions do not show significant changes in expression (2p22.p21 comprising MSH2, 2p16 comprising MSH6, 3p22 comprising TGFBR2). Eleven further chromosomal regions are linked to hereditary colorectal carcinoma under a common entry in OMIM (14500). More than 50% of these regions show significant expression changes in our data. Five regions show down-regulation (1p35, 14q24.3, 17p11.2, 17p13.1, 22q13), one region shows up-regulation (2p25), and five regions do not show significant expression changes in our data (3q26.3, 8p22-p21.3, 11p11.2, 15q15, 17q24). In combination, these findings strongly suggest that expression changes in regions linked to hereditary CRC play a role in CRC development.

### Congruence of our study with the genome-wide copy number and expression analysis of Tsafrir et al

A particular focus of our study was on the congruence of our data with that of Tsafrir et al. [17]. These authors described 11 alterations of whole chromosomes or chromosome arms. Using our approach based solely on expression data we found precisely defined region of coordinated up-regulation in all four regions of gene expression and gDNA copy number gain that they reported (+7, +8q, +13q, +20q). For six of seven aberrations (-1p, -4, -5q, -14q, -15q, -18) we discovered smaller expression islands of coordinated down-regulation. We were not able to reproduce the finding of expression loss on 8p. In summary, this large congruence of our results with that of Tsafrir et al. can be regarded as an external validation of our results. The comparison illustrates the power of our data analysis approach which allows to define expression islands on a single-gene resolution. Most importantly it confirms our confidence in the use of the chip platform (Affymetrix U133A) that was used in both studies and apparently can lead to largely congruent results in different patient cohorts and laboratories.

## Conclusion

Roughly a quarter of all human genes is located in islands of misregulated gene expression in colorectal cancer. There are only slightly more down-regulated than up-regulated genes. Chromosomal regions that are linked to hereditary colorectal cancer often exhibit deregulated expression, suggesting that they are implicated in spontaneous CRC not only through collection of mutations. Thus, genes in these chromosomal hotspots may be systematically tested in patients with sporadic CRC for molecular lesions and for transcriptional silencing.

Chromosomal regions that are frequently deleted in CRC very often comprise islands in which we found reduced expression. Although many regions that are known to be amplified in colorectal tumors show a gain of expression, there are also a considerable number of amplified islands that show no alterations or even down-regulation. Comparison of published CGH studies with our expression data suggests that amplified or deleted chromosomal regions are responsible for many islands with aberrant expression. However, we suggest that it is necessary to invoke other mechanism like epigenetic regulation of chromatin or disruption of enhancer actions to explain the remaining expression imbalances.

## Methods

### Patients

25 colorectal cancer patients undergoing elective standard oncological resection at the department of surgery, Charité, Campus Benjamin Franklin, Berlin, Germany were prospectively recruited for this study. The study was approved by the local ethical committee and informed consent was obtained from all patients. Rectal cancer patients receiving neo-adjuvant radiochemotherapy were excluded from this study.

### Tissue samples and UV-laser microdissection

Transmural cancer specimens were snap frozen (liquid nitrogen) within 20 minutes following excision and stored at -80°C. All tissue samples were evaluated by a pathologist before and during laser micro-dissection to ensure an enrichment of vital tumor cells. Six-micron serial frozen sections were cut on a standard cryostat and mounted on RNase-free foil (2,5 μm) coated on glass slides followed by immediate fixation (70% ethanol for 30s), H&E staining, and ethanol dehydration (70%, 95% and finally 100% ethanol). After vacuum drying the membranes carrying the sections were manually turned and coated on new RNase free glass slides. Optically transparent CapSure LCM caps (ARCTURUS, CA) were placed on the foil over a selected field of cells. Vital colorectal epithelial carcinoma cells (> 90% proportion) from the invasion front were isolated using UV-LCM Systems from PALM (Microlaser Technologie, Germany) and SL (Microtest GmbH, Germany). After visual control of completeness of dissection the captured cells were immersed in denaturation buffer (GTC Extraction Buffer, 2% beta-mercaptoethanol, Promega, WI) and stored at -80°C.

### mRNA-extraction, cRNA-preparation and -amplification

Poly(A)+ RNAs were isolated using PolyATtract 1000 kit (Promega, Heidelberg, Germany) according to the manufacturer's recommendations. For each sample the cDNA synthesis and repetitive *in vitro *transcription were performed three times, as described previously [38-40]. In brief, the total amount of prepared mRNA from one sample was used. First strand cDNA synthesis was initiated using the Affymetrix T7-oligo-dT promoter-primer combination. The second strand cDNA was synthesized by internal priming. *In vitro *transcription was performed using Ambion's Megascript kit (Ambion, Huntington, UK) as recommended by the manufacturer. From the generated cRNA a new first strand synthesis was initiated using 0.025 mM of a random hexamer as primer. After completion, the second strand synthesis was primed using the Affymetrix T7-oligo-dT promoter-primer combination at a concentration of 0.1 mM. A second *in vitro *transcription was performed and then the procedure was repeated one additional time. During the third *in vitro *transcription biotin-labeled nucleotides were incorporated into the cRNA as recommended by the Affymetrix protocol.

### Microarray hybridization

BIO+cRNAs were hybridized on Affymetrix Human Genome U133A and U133B GeneChips, that consist of 44.928 probe sets (Affymetrix, Santa Clara, CA). Fragmentation, preparation of hybridization cocktails, hybridization, washing, staining and scanning of Affymetrix GeneChip were performed according to the manufacturer's protocols.

### Preprocessing of expression data

We used our own algorithm to condensate the probe level data provided by Affymetrix CEL-files per chip experiment: Background intensity was computed as the mean of the 2% darkest feature intensities. This background value was subtracted from each feature value. Subsequently, each feature value was divided by the median of all feature values. As a representative expression value (PMQ) for each probe set, the third quartile (75%) of all intensities of all perfect match oligonucleotides was used. Furthermore, to distinguish real expression signals from noise the Wilcoxon signed rank test was applied to each probe set. A probe set was called detectable if the result of the Wilcoxon signed rank test applied to its 11 probe pairs (perfect match versus mismatch oligonucleotide) had a significance level of p < 0.1 and relative expression value (PMQ) of > 4.0. We used these constraints for decision whether a gene is expressed or not due to validation results of several gene expression pattern by quantitative RT-PCR and/or Northern Blot analysis in our lab (data not shown).

For each patient and probeset an expression ratio was calculated according to the following rules: If expression was detectable in both the normal and tumor sample (Wilcoxon test p <= 0.10 and relative expression value PMQ >= 4), the ratio PMQ(T)/PMQ(N) is our expression ratio (hereafter called T/N). If expression was undetectable in either the normal or the tumor sample, the expression ratio was either set to T/N = 2 (normal absent) or to T/N = 0.5 (tumor absent). If expression was undetectable in both the normal and tumor sample, no expression ratio was calculated and we call the probe set not informative. For each probe set the number of cases which showed an up-regulation (T/N >= 2), a down-regulation (T/N <= 0.5) or the number of unchanged transcription levels (0.5 < T/N < 2) were counted. We filtered out those probe sets which are not informative in any patient, reducing the number of probe sets to 19404. To eliminate redundancy of probe sets with respect to genes, we kept only the most informative probe set of a single gene, i.e. the probe set which is informative in the highest number of matched sample pairs. Additionally, only probe sets that could unambiguously be linked to a particular genomic locus were considered (chromosome band and position; see Affymetrix U133A/B annotation files). Finally, the pre-processing resulted in a total number of 10.935 probe sets which were the basis of all further analyses.

### Analysis of expression along chromosomes

In each graph of Figures 2, 3, 4, 5, we plotted the numbers of patient samples with tumor up/down regulation (percentage on informative cases) for all genes according to their position on the chromosome. In these plots, the smoothing of the curve is achieved by averaging over 50 consecutive genes.

Significant deviations from average expression in a particular chromosomal region is not sufficient to infer coordinated deregulation. This is because it does not allow to infer whether all genes of a region are actually de-regulated in the same subset of patients. They could also be de-regulated in different patients. Consider three genes G1, G2, G3 and their expression in patients A,B,C,D. Each gene is up-regulated in 50% of patients. If the genes are up-regulated in different patients (G1 is up-regulated in A/B, G2 is up-regulated in B/C, G3 is up-regulated in C/D), then one can not assume that there is a regional up-regulation in all patients. However, if the genes are up-regulated in the same patients (G1, G2 and G3 are all up-regulated in A and B), then it is fair to assume that they have undergone coordinated regional up-regulation. Chance effects more likely create non-coordinated up-regulation. To capture such a gene-versus-gene correlation structure, we performed the following for a given chromosome region:

For each pair of genes of a given chromosome region we count the number of their coordinated (simultaneous) up-regulations (based on the above computed fold changes) over the set of patients and the number of coordinated down-regulations, separately. These values can be represented in gray-scale plots: one gray scale plot for the coordinated up-regulation and a similar one for coordinated down-regulation. Both, horizontal and vertical axis comprise genes of the chromosome region in the right chromosomal order (see Figures 6, 7, 8, 9, 10, 11, 12, 13, 14, 15, 16, 17, 18, 19, 20, 21, 22, 23, 24, 25, 26, 27, 28, 29, 30, 31, 32). The darkness of squares represents the number of coordinated up- or down-regulations, respectively. Coordinately up-regulated regions show up as squares with high "correlation" measures along the diagonal. Such resulting cross-comparison matrices can be visualized interactively for any chromosomal region on our supplementary website[41] along with heat maps of expression intensities and are used in Figures 6, 7, 8, 9, 10, 11, 12, 13, 14, 15, 16, 17, 18, 19, 20, 21, 22, 23, 24, 25, 26, 27, 28, 29, 30, 31, 32. Alternatively, we applied "correlation" measures like Pearson correlation coefficients on fold changes, mutual information, and set-theoretic coefficients like the Dice and Jaccard coefficients on binary patterns of up-regulation and down-regulation (only available on our website [41]).

Although this analysis is already instructive for the visual identification of general up/down-regulation of a particular region, it does not allow to infer the precise boundaries of deregulated regions. Several software packages for the analysis of array CGH data exist that have been announced to also be suited for the analysis of expression data [42-44]. In the following, we used the ChARM software package [44]. ChARM can be used to infer intervals of variable size with significant positive or negative signal amplitudes in ordered data, such as log(intensity) values in array CGH data and mRNA expression data. We applied the ChARM algorithm on different data sets that harbor information about the numbers of patients with coordinated up- and down-regulation of expression for all genes on human autosomes and the X chromosome. For each chromosome six separate data sets were prepared, according to scanning window sizes of 5, 11, 21, 31, 41, 51. Within each window all possible gene pairs (excluding self comparisons) were considered. For each gene pair, the number of coordinated up-regulated (counted as +1) and down-regulated (counted as -1) was determined. For each window the sum of these gene pair-specific values divided by the total number of pairs gave the cumulative misregulation score (CMS). In a sliding window approach, each gene was associated with a CMS value. CMS values for genes at the edges of chromosomes were calculated with reduced window sizes. The main theoretical advantage of the use of CMS scores compared to raw up-regulation counts or averaged expression ratios is that it captures only information from co-regulated neighboring gene pairs: Noise signals fluctuate across genes and may more often lead to artificial assignment of high expression ratios between two genes. In contrast, real signals of regional up-/down-regulation lead to consistent changes in the same patients for two genes. For each window size, CMS data sets of each chromosome were subject to ChARM analysis. ChARM determines borders of regions with high signal amplitudes in ordered data, here regions of expression imbalances along a chromosome, by an expectation-maximization approach. In addition, ChARM provides different statistical estimates to judge the significance of expression deregulation in a particular chromosomal region [44]. The identified deregulated regions were further evaluated manually using heat maps and the above mentioned gene-versus-gene "correlation" plots (see above, Figures 6, 7, 8, 9, 10, 11, 12, 13, 14, 15, 16, 17, 18, 19, 20, 21, 22, 23, 24, 25, 26, 27, 28, 29, 30, 31, 32 and accompanying website).

### Availability and requirements

Project name: Colorectal carcinoma comparative chromosomal gene expression analysis (CC-CCGEA) [41].

Project home page: 

Operating system(s): all

Programming language: Perl-CGI

Licence: GNU GPL

Restrictions to use by non-academics: none

## Competing interests

The author(s) declare that they have no competing interests.

## Authors' contributions

ES guided and performed data analysis and drafted the manuscript, JG performed RNA sample preparation, microdissection and hybridization of chips, DM hybridized DNA chips and contributed to data analysis, SR was involved in data preprocessing and analysis, IK performed laser capture microdissection, ECV did laser capture microdissection, TB supervised chip hybridization and data preprocessing, BM and HJB were responsible for clinical part of the study including sample acquisition and patients' informed consent, CP and BW supervised chip hybridization, quality control and data preprocessing, AR conceived the study and revised the manuscript.

## Supplementary Material

Additional file 1 original ChARM output on chromosomal intervals of coordinated up- or down-regulated expression. This files contains the full original output of ChARM analyses (see methods section). Annotation of probeset IDs with gene symbols and chromosome bands was added subsequently. Click here for file
