# Supplementary material for: A genome-wide map of aberrantly expressed chromosomal islands in colorectal cancer
Source: Mol Cancer. 2006 Sep 18;5:37. doi: 10.1186/1476-4598-5-37 (PMC1601966; doi:10.1186/1476-4598-5-37)
Supplement: Additional file 1 — original ChARM output on chromosomal intervals of coordinated up- or down-regulated expression. This files contains the full original output of ChARM analyses (see methods section). Annotation of probeset IDs with gene symbols and chromosome bands was added subsequently. [file 1476-4598-5-37-S1.doc]

| Experiment | Chromosome | Start ID | Start ChrBand | Start GeneSym | Start ChrPos | End ID | End ChrBand | End GeneSym | End ChrPos | Mean value over region | Sign test (positive) p-value | Sign test (negative) p-value | Mean test p-value |
| --- | --- | --- | --- | --- | --- | --- | --- | --- | --- | --- | --- | --- | --- |
| CNT_COMBINED_SL5 | 1 | 219739_AT_HG-U133A | chr1p36.13 | FLJ20225 | 19610081 | 226272_AT_HG-U133B | chr1p36.11 | Hs.22380.1 | 24218138 | -2.010526323 | 1 | 1.45E-06 | 4.84E-14 |
| CNT_COMBINED_SL5 | 16 | 219395_AT_HG-U133A | chr16q22.1 | FLJ21918 | 68039437 | 226072_AT_HG-U133B | chr16q22.1 | FUK | 70289789 | 1.280769221 | 5.80E-04 | 1 | 2.99E-04 |
| CNT_COMBINED_SL5 | 23 | 228262_AT_HG-U133B | chrXp22.12 | FLJ14503 | 19386473 | 238878_AT_HG-U133B | chrXp22.11 | ARX | 24383281 | 1.72941177 | 6.33E-04 | 1 | 0.006391661 |
| CNT_COMBINED_SL5 | 5 | 203024_S_AT_HG-U133A | chr5q31.1 | HTGN29 | 133367495 | 222984_AT_HG-U133B | chr5q31.2 | PAIP2 | 138781064 | 0.74210527 | 5.61E-04 | 1 | 0.002942947 |
| CNT_COMBINED_SL5 | 5 | 226982_AT_HG-U133B | chr5q15 | ELL2 | 95294929 | 218706_S_AT_HG-U133A | chr5q23.2 | NS3TP2 | 125905260 | -0.6882353 | 1 | 9.50E-04 | 6.54E-06 |
| CNT_COMBINED_SL5 | 7 | 236529_AT_HG-U133B | chr7q11.23 | SRCRB4D | 75630814 | 205542_AT_HG-U133A | chr7q21.13 | STEAP | 89402598 | -1.081818166 | 1 | 0.008300781 | 2.74E-06 |
| CNT_COMBINED_SL11 | 1 | 228096_AT_HG-U133B | chr1p36.13 | Hs.15768.0 | 19425206 | 202838_AT_HG-U133A | chr1p36.11 | FUCA1 | 23641181 | -1.693366089 | 1 | 1.03E-06 | 0 |
| CNT_COMBINED_SL11 | 1 | 230660_AT_HG-U133B | chr1q32.2 | DJ667H12.2 | 207498659 | 217894_AT_HG-U133A | chr1q41 | KCTD3 | 212850982 | 1.177272724 | 3.10E-04 | 1 | 1.94E-09 |
| CNT_COMBINED_SL11 | 11 | 206050_S_AT_HG-U133A | chr11p15.5 | RNH | 484560 | 213432_AT_HG-U133A | chr11p15.5 | MUC5AC | 1249809 | -1.069318192 | 1 | 0.006347656 | 6.11E-08 |
| CNT_COMBINED_SL11 | 12 | 201600_AT_HG-U133A | chr12p13.31 | REA | 6944845 | 219761_AT_HG-U133A | chr12p13.2 | CLEC1 | 10114395 | -0.718716578 | 1 | 0.001108494 | 8.57E-06 |
| CNT_COMBINED_SL11 | 12 | 218102_AT_HG-U133A | chr12p12.3 | CGI-26 | 16080887 | 227416_S_AT_HG-U133B | chr12q12 | MADP-1 | 40993994 | -0.301983472 | 1 | 0.004272461 | 4.32E-04 |
| CNT_COMBINED_SL11 | 12 | 219937_AT_HG-U133A | chr12q21.1 | TRHDE | 71345170 | 201603_AT_HG-U133A | chr12q21.2 | PPP1R12A | 78670123 | 1.933884285 | 0.008544922 | 1 | 0 |
| CNT_COMBINED_SL11 | 12 | 223315_AT_HG-U133B | chr12q22 | NTN4 | 94554124 | 203743_S_AT_HG-U133A | chr12q23.3 | TDG | 102884514 | 0.635016833 | 6.54E-04 | 1 | 0.001648371 |
| CNT_COMBINED_SL11 | 13 | 223306_AT_HG-U133B | chr13q14.2 | EBRP | 48032936 | 228915_AT_HG-U133B | chr13q21.33 | DACH | 69810172 | 2.973484844 | 5.81E-05 | 1 | 3.82E-09 |
| CNT_COMBINED_SL11 | 14 | 201699_AT_HG-U133A | chr14q22.1 | PSMC6 | 51177917 | 204728_S_AT_HG-U133A | chr14q22.2 | AND-1 | 53397678 | 1.120661167 | 0.006347656 | 1 | 1.07E-12 |
| CNT_COMBINED_SL11 | 16 | 219575_S_AT_HG-U133A | chr16q22.1 | COG8 | 69138788 | 201000_AT_HG-U133A | chr16q22.1 | AARS | 70062578 | 2.086363643 | 0.004150391 | 1 | 2.96E-08 |
| CNT_COMBINED_SL11 | 16 | 224830_AT_HG-U133B | chr16q12.2 | CPSF5 | 56238610 | 218060_S_AT_HG-U133A | chr16q13 | FLJ13154 | 57830335 | -2.1219697 | 1 | 0.004150391 | 2.52E-11 |
| CNT_COMBINED_SL11 | 16 | 40149_AT_HG-U133A | chr16p11.2 | SH2B | 28922704 | 212046_X_AT_HG-U133A | chr16p11.2 | MAPK3 | 30162793 | -1.624242417 | 1 | 0.001076822 | 9.19E-04 |
| CNT_COMBINED_SL11 | 17 | 204206_AT_HG-U133A | chr17p13.3 | MNT | 2493952 | 226262_AT_HG-U133B | chr17p13.2 | DHX33 | 5544815 | -0.918403549 | 1 | 4.27E-06 | 3.63E-09 |
| CNT_COMBINED_SL11 | 17 | 228834_AT_HG-U133B | chr17q21.33 | TOB1 | 49415550 | 200614_AT_HG-U133A | chr17q23.2 | CLTC | 58246089 | 1.284029477 | 6.92E-08 | 1 | 0 |
| CNT_COMBINED_SL11 | 18 | 224629_AT_HG-U133B | chr18q21.32 | LMAN1 | 55144128 | 227072_AT_HG-U133B | chr18q22.2 | RTTN | 65820176 | -2.647933873 | 1 | 0.002929688 | 0 |
| CNT_COMBINED_SL11 | 18 | 226866_AT_HG-U133B | chr18q11.2 | KIAA1911 | 17361296 | 203726_S_AT_HG-U133A | chr18q11.2 | LAMA3 | 19784937 | -1.82419914 | 1 | 0.005859375 | 1.36E-04 |
| CNT_COMBINED_SL11 | 2 | 226863_AT_HG-U133B | chr2p25.3 | Hs.8379.0 | 28878 | 216640_S_AT_HG-U133A | chr2p25.1 | Hs.182429.2 | 10946010 | 0.892597859 | 2.28E-04 | 1 | 9.55E-07 |
| CNT_COMBINED_SL11 | 20 | 209061_AT_HG-U133A | chr20q13.12 | NCOA3 | 46970510 | 213405_AT_HG-U133A | chr20q13.32 | Hs.512358 | 57627450 | 2.029090901 | 6.31E-06 | 1 | 4.88E-15 |
| CNT_COMBINED_SL11 | 22 | 209138_X_AT_HG-U133A | chr22q11.22 | IGL@ | 21490269 | 54037_AT_HG-U133A | chr22q12.1 | HPS4 | 25172642 | -1.768939381 | 1 | 6.02E-06 | 0 |
| CNT_COMBINED_SL11 | 22 | 238058_AT_HG-U133B | chr22q13.31 | Hs.296370.0 | 44661912 | 219151_S_AT_HG-U133A | chr22q13.33 | RABL2B | 49338558 | -1.110389614 | 1 | 1.37E-04 | 9.83E-05 |
| CNT_COMBINED_SL11 | 23 | 232087_AT_HG-U133B | chrXp22.12 | LOC256643 | 19295853 | 204835_AT_HG-U133A | chrXp22.11 | POLA | 24375933 | 1.671657745 | 6.65E-04 | 1 | 0 |
| CNT_COMBINED_SL11 | 5 | 201506_AT_HG-U133A | chr5q31.1 | TGFBI | 135475142 | 229167_AT_HG-U133B | chr5q31.3 | Hs.443121 | 139526902 | 0.690909087 | 2.68E-04 | 1 | 0.002924977 |
| CNT_COMBINED_SL11 | 5 | 225698_AT_HG-U133B | chr5q22.1 | TIGA1 | 111572888 | 219511_S_AT_HG-U133A | chr5q23.2 | SNCAIP | 121875446 | -0.950802133 | 1 | 0.002502441 | 2.80E-07 |
| CNT_COMBINED_SL11 | 6 | 205671_S_AT_HG-U133A | chr6p21.32 | HLA-DOB | 32827567 | 201049_S_AT_HG-U133A | chr6p21.32 | RPS18 | 33287291 | -0.99431818 | 1 | 0.002319336 | 1.21E-10 |
| CNT_COMBINED_SL11 | 8 | 242763_AT_HG-U133B | chr8q23.1 | Hs.157897 | 110468924 | 212149_AT_HG-U133A | chr8q24.22 | KIAA0143 | 132982022 | 1.115530311 | 1.94E-06 | 1 | 0 |
| CNT_COMBINED_SL21 | 1 | 201209_AT_HG-U133A | chr1p35.1 | HDAC1 | 32301335 | 200039_S_AT_HG-U133B | chr1p34.3 | PSMB2 | 35496439 | -0.802040813 | 1 | 1.12E-04 | 0.002332483 |
| CNT_COMBINED_SL21 | 1 | 208783_S_AT_HG-U133A | chr1q32.2 | MCP | 205051907 | 229376_AT_HG-U133B | chr1q32.3 | PROX1 | 211270329 | 1.053209103 | 6.64E-05 | 1 | 0 |
| CNT_COMBINED_SL21 | 1 | 211165_X_AT_HG-U133A | chr1p36.12 | EPHB2 | 22706409 | 209007_S_AT_HG-U133A | chr1p36.11 | DJ465N24.2.1 | 24919756 | -1.433509707 | 1 | 8.34E-06 | 0 |
| CNT_COMBINED_SL21 | 1 | 225920_AT_HG-U133B | chr1p36.33 | LOC148413 | 1288307 | 209354_AT_HG-U133A | chr1p36.32 | TNFRSF14 | 2358801 | -0.94920634 | 1 | 0.002597042 | 0.006678632 |
| CNT_COMBINED_SL21 | 10 | 241222_AT_HG-U133B | chr10p15.3 | Hs.371616 | 2224830 | 227210_AT_HG-U133B | chr10p14 | SFMBT2 | 7204744 | -0.694057365 | 1 | 0.001171836 | 1.79E-04 |
| CNT_COMBINED_SL21 | 11 | 203947_AT_HG-U133A | chr11p13 | CSTF3 | 33070519 | 205558_AT_HG-U133A | chr11p12 | TRAF6 | 36475400 | 0.576190485 | 1.82E-04 | 1 | 3.19E-04 |
| CNT_COMBINED_SL21 | 11 | 204960_AT_HG-U133A | chr11q13.2 | PTPRCAP | 66978365 | 229250_AT_HG-U133B | chr11q13.3 | TPCN2 | 68630877 | -0.733571433 | 1 | 1.08E-04 | 5.30E-09 |
| CNT_COMBINED_SL21 | 12 | 203824_AT_HG-U133A | chr12q15 | TM4SF3 | 69805228 | 229018_AT_HG-U133B | chr12q21.31 | FLJ22789 | 81352972 | 1.141190466 | 8.50E-05 | 1 | 0 |
| CNT_COMBINED_SL21 | 12 | 209128_S_AT_HG-U133A | chr12q23.3 | SART3 | 107419000 | 226583_AT_HG-U133B | chr12q24.11 | Hs.18370.0 | 108941840 | -0.469576725 | 1 | 0.00112915 | 2.11E-04 |
| CNT_COMBINED_SL21 | 12 | 227439_AT_HG-U133B | chr12q23.1 | EB-1 | 97640996 | 212795_AT_HG-U133A | chr12q23.3 | KIAA1033 | 104064827 | 0.593197283 | 8.50E-05 | 1 | 5.16E-05 |
| CNT_COMBINED_SL21 | 12 | 233149_AT_HG-U133B | chr12q14.2 | Hs.132260.0 | 63076549 | 222845_X_AT_HG-U133B | chr12q14.3 | CGI-119 | 64818025 | 0.628571432 | 0.009033203 | 1 | 7.65E-04 |
| CNT_COMBINED_SL21 | 13 | 227415_AT_HG-U133B | chr13q14.11 | LOC283508 | 40614512 | 212402_AT_HG-U133A | chr13q14.13 | KIAA0853 | 44328648 | 2.639583349 | 0.001098633 | 1 | 2.99E-09 |
| CNT_COMBINED_SL21 | 13 | 235283_AT_HG-U133B | chr13q14.3 | Hs.512272 | 49734562 | 213239_AT_HG-U133A | chr13q22.1 | PIBF1 | 71337502 | 2.914512464 | 4.13E-05 | 1 | 0 |
| CNT_COMBINED_SL21 | 14 | 240632_AT_HG-U133B | chr14q24.1 | Hs.369329 | 66929910 | 229297_AT_HG-U133B | chr14q24.3 | Hs.169812 | 74667609 | -0.576937448 | 1 | 2.66E-08 | 0 |
| CNT_COMBINED_SL21 | 15 | 218396_AT_HG-U133A | chr15q22.2 | FLJ10381 | 59860680 | 235689_AT_HG-U133B | chr15q22.31 | MtFMT | 63011271 | -1.138961038 | 1 | 9.68E-05 | 0 |
| CNT_COMBINED_SL21 | 15 | 227008_AT_HG-U133B | chr15q26.1 | Hs.349979 | 89203946 | 228954_AT_HG-U133B | chr15q26.3 | FLJ33008 | 98002978 | -1.083458646 | 1 | 9.68E-05 | 4.33E-13 |
| CNT_COMBINED_SL21 | 15 | 239413_AT_HG-U133B | chr15q21.1 | KIAA0912 | 46746220 | 202603_AT_HG-U133A | chr15q21.3 | ADAM10 | 56603779 | -1.117414961 | 1 | 7.17E-06 | 0 |
| CNT_COMBINED_SL21 | 16 | 201131_S_AT_HG-U133A | chr16q22.1 | CDH1 | 68645053 | 212189_S_AT_HG-U133A | chr16q22.1 | COG4 | 70290676 | 1.357857144 | 7.81E-05 | 1 | 4.15E-14 |
| CNT_COMBINED_SL21 | 16 | 227889_AT_HG-U133B | chr16q12.2 | CAPNS2 | 55398720 | 204519_S_AT_HG-U133A | chr16q13 | TM4SF11 | 57065455 | -1.792619044 | 1 | 7.81E-05 | 4.11E-12 |
| CNT_COMBINED_SL21 | 16 | 35671_AT_HG-U133A | chr16p12.1 | GTF3C1 | 27438333 | 228513_AT_HG-U133B | chr16p11.2 | LOC124446 | 30021453 | -1.368333334 | 1 | 7.81E-05 | 2.12E-06 |
| CNT_COMBINED_SL21 | 17 | 203721_S_AT_HG-U133A | chr17q21.33 | CGI-48 | 49837322 | 227020_AT_HG-U133B | chr17q23.2 | Hs.368672 | 57953151 | 1.275238099 | 6.71E-07 | 1 | 0 |
| CNT_COMBINED_SL21 | 17 | 209141_AT_HG-U133A | chr17p13.2 | UBE2G1 | 4381352 | 202900_S_AT_HG-U133A | chr17p13.2 | NUP88 | 5490598 | -0.790714297 | 1 | 2.01E-04 | 1.12E-04 |
| CNT_COMBINED_SL21 | 18 | 235022_AT_HG-U133B | chr18p11.21 | MGC24180 | 13655709 | 206032_AT_HG-U133A | chr18q12.1 | DSC3 | 26822394 | -1.169917858 | 1 | 0.001831055 | 0.001385019 |
| CNT_COMBINED_SL21 | 19 | 205382_S_AT_HG-U133A | chr19p13.3 | DF | 812901 | 223348_X_AT_HG-U133B | chr19p13.3 | MUM-1 | 1324081 | -0.63699634 | 1 | 0.002319336 | 2.20E-05 |
| CNT_COMBINED_SL21 | 19 | 209594_X_AT_HG-U133A | chr19q13.31 | PSG9 | 48454191 | 227689_AT_HG-U133B | chr19q13.31 | ZNF227 | 49432829 | -0.704329003 | 1 | 0.009277344 | 4.08E-06 |
| CNT_COMBINED_SL21 | 19 | 220135_S_AT_HG-U133A | chr19q13.11 | SLC7A9 | 38013344 | 202475_AT_HG-U133A | chr19q13.12 | NIFIE14 | 40729301 | 0.456987576 | 1.47E-04 | 1 | 1.64E-08 |
| CNT_COMBINED_SL21 | 2 | 202506_AT_HG-U133A | chr2q31.3 | SSFA2 | 182997199 | 209272_AT_HG-U133A | chr2q32.2 | NAB1 | 191759464 | 0.742355889 | 1.94E-04 | 1 | 1.31E-05 |
| CNT_COMBINED_SL21 | 2 | 228020_AT_HG-U133B | chr2p11.2 | FLJ20758 | 86342728 | 213403_AT_HG-U133A | chr2q12.1 | MGC11332 | 102952522 | -0.450695128 | 1 | 4.87E-04 | 0 |
| CNT_COMBINED_SL21 | 2 | 235360_AT_HG-U133B | chr2q33.3 | Hs.87980.0 | 208888585 | 201511_AT_HG-U133A | chr2q35 | AAMP | 219331507 | 0.713784467 | 3.27E-04 | 1 | 0.001881705 |
| CNT_COMBINED_SL21 | 2 | 235673_AT_HG-U133B | chr2p25.1 | Hs.262864.0 | 10099067 | 231439_AT_HG-U133B | chr2p24.3 | Hs.110039.0 | 14801501 | 1.121587296 | 0.001583562 | 1 | 2.38E-10 |
| CNT_COMBINED_SL21 | 20 | 208720_S_AT_HG-U133A | chr20q11.22 | RNPC2 | 35007698 | 226973_AT_HG-U133B | chr20q11.23 | C20orf102 | 37258623 | 1.90306122 | 7.02E-04 | 1 | 5.20E-11 |
| CNT_COMBINED_SL21 | 20 | 222518_AT_HG-U133B | chr20q13.13 | ARFGEF2 | 48337397 | 211678_S_AT_HG-U133A | chr20q13.13 | MEG3 | 49255560 | 2.170995691 | 0.005615234 | 1 | 5.11E-15 |
| CNT_COMBINED_SL21 | 21 | 237923_AT_HG-U133B | chr21q22.13 | Hs.146186 | 37004028 | 226553_AT_HG-U133B | chr21q22.3 | TMPRSS2 | 41757027 | 0.445039688 | 2.22E-04 | 1 | 1.11E-08 |
| CNT_COMBINED_SL21 | 22 | 203878_S_AT_HG-U133A | chr22q11.23 | MMP11 | 22450471 | 227014_AT_HG-U133B | chr22q12.1 | LOC57168 | 25163701 | -1.661904772 | 1 | 6.02E-04 | 0 |
| CNT_COMBINED_SL21 | 22 | 33778_AT_HG-U133A | chr22q13.31 | C22orf4 | 45781828 | 219151_S_AT_HG-U133A | chr22q13.33 | RABL2B | 49338558 | -1.146015139 | 1 | 2.10E-04 | 1.24E-07 |
| CNT_COMBINED_SL21 | 23 | 218793_S_AT_HG-U133A | chrXp22.13 | SCML1 | 17133929 | 214678_X_AT_HG-U133A | chrXp22.11 | ZFX | 23593530 | 1.108163269 | 4.67E-04 | 1 | 9.76E-07 |
| CNT_COMBINED_SL21 | 23 | 241093_AT_HG-U133B | chrXq24 | Hs.178536 | 117510187 | 218853_S_AT_HG-U133A | chrXq26.3 | DJ473B4 | 132727373 | 1.444257701 | 5.40E-07 | 1 | 0 |
| CNT_COMBINED_SL21 | 3 | 204294_AT_HG-U133A | chr3p21.31 | AMT | 49413272 | 206855_S_AT_HG-U133A | chr3p21.31 | HYAL2 | 50314302 | -0.640952385 | 1 | 0.004882813 | 4.05E-05 |
| CNT_COMBINED_SL21 | 3 | 212399_S_AT_HG-U133A | chr3p25.3 | KIAA0121 | 11572585 | 203356_AT_HG-U133A | chr3p25.1 | CAPN7 | 15268230 | 0.517006809 | 4.42E-04 | 1 | 0.001004729 |
| CNT_COMBINED_SL21 | 3 | 219133_AT_HG-U133A | chr3p24.2 | FLJ20604 | 25808356 | 212309_AT_HG-U133A | chr3p23 | CLASP2 | 33510295 | 0.541854647 | 0.00126685 | 1 | 1.05E-04 |
| CNT_COMBINED_SL21 | 3 | 219510_AT_HG-U133A | chr3q13.33 | POLQ | 122471228 | 201125_S_AT_HG-U133A | chr3q21.2 | ITGB5 | 125802773 | -0.464285719 | 1 | 0.00126685 | 5.60E-04 |
| CNT_COMBINED_SL21 | 3 | 226524_AT_HG-U133B | chr3p11.1 | MGC26717 | 88126398 | 223218_S_AT_HG-U133B | chr3q12.3 | MAIL | 102900236 | 0.922769001 | 3.24E-05 | 1 | 0 |
| CNT_COMBINED_SL21 | 4 | 202825_AT_HG-U133A | chr4q35.1 | SLC25A4 | 186762837 | 242822_AT_HG-U133B | chr4q35.2 | Hs.130535 | 191169554 | -1.084717888 | 1 | 0.001953125 | 0 |
| CNT_COMBINED_SL21 | 4 | 212455_AT_HG-U133A | chr4q13.2 | YT521 | 69182470 | 206336_AT_HG-U133A | chr4q13.3 | CXCL6 | 75170216 | -0.86054422 | 1 | 0.0078125 | 2.62E-06 |
| CNT_COMBINED_SL21 | 4 | 217933_S_AT_HG-U133A | chr4p15.32 | LAP3 | 17359917 | 240095_AT_HG-U133B | chr4p15.2 | Hs.129636 | 24556082 | -0.917346937 | 1 | 9.77E-04 | 3.82E-09 |
| CNT_COMBINED_SL21 | 5 | 209788_S_AT_HG-U133A | chr5q15 | ARTS-1 | 96184289 | 215446_S_AT_HG-U133A | chr5q23.1 | Hs.102267.3 | 121476642 | -0.667380957 | 1 | 1.80E-07 | 0 |
| CNT_COMBINED_SL21 | 5 | 226742_AT_HG-U133B | chr5q31.1 | Hs.432984 | 134013101 | 218755_AT_HG-U133A | chr5q31.2 | KIF20A | 137599032 | 0.728822052 | 1.51E-04 | 1 | 8.32E-05 |
| CNT_COMBINED_SL21 | 5 | 237100_AT_HG-U133B | chr5q32 | Hs.184323 | 147120427 | 224068_X_AT_HG-U133B | chr5q33.1 | FLJ10290 | 150099231 | -0.589393945 | 1 | 0.001190186 | 8.72E-07 |
| CNT_COMBINED_SL21 | 6 | 212998_X_AT_HG-U133A | chr6p21.32 | HLA-DQB2 | 32673757 | 209110_S_AT_HG-U133A | chr6p21.32 | RAB2L | 33306354 | -0.782086174 | 1 | 8.78E-04 | 0 |
| CNT_COMBINED_SL21 | 6 | 218845_AT_HG-U133A | chr6p25.3 | DUSP22 | 295820 | 202149_AT_HG-U133A | chr6p24.2 | NEDD9 | 11291573 | 0.707350022 | 9.96E-09 | 1 | 0 |
| CNT_COMBINED_SL21 | 6 | 228069_AT_HG-U133B | chr6q23.3 | DUFD1 | 136532766 | 223361_AT_HG-U133B | chr6q24.1 | PRO2013 | 139344505 | 0.789915958 | 0.001488548 | 1 | 0.003295651 |
| CNT_COMBINED_SL21 | 7 | 201889_AT_HG-U133A | chr7q31.31 | FAM3C | 120543503 | 220949_S_AT_HG-U133A | chr7q33 | MGC5242 | 134266664 | 0.691957672 | 3.40E-05 | 1 | 2.48E-12 |
| CNT_COMBINED_SL21 | 7 | 204202_AT_HG-U133A | chr7p22.3 | KIAA1023 | 2398122 | 242345_AT_HG-U133B | chr7p21.3 | Hs.170023.0 | 7142548 | 0.972817463 | 2.02E-05 | 1 | 2.57E-10 |
| CNT_COMBINED_SL21 | 7 | 228766_AT_HG-U133B | chr7q21.11 | CD36 | 79920225 | 219342_AT_HG-U133A | chr7q21.3 | CAS1 | 93797733 | -0.874747473 | 1 | 9.64E-05 | 0 |
| CNT_COMBINED_SL21 | 8 | 238017_AT_HG-U133B | chr8q12.1 | RDH-E2 | 57263867 | 225547_AT_HG-U133B | chr8q13.2 | Hs.372680 | 67884213 | 0.850103513 | 2.59E-05 | 1 | 2.52E-06 |
| CNT_COMBINED_SL21 | 8 | 241342_AT_HG-U133B | chr8q24.13 | LOC157378 | 125280157 | 203760_S_AT_HG-U133A | chr8q24.22 | SLA | 134006556 | 1.227437632 | 1.24E-04 | 1 | 0 |
| CNT_COMBINED_SL21 | 8 | 244447_AT_HG-U133B | chr8q22.3 | Hs.435001 | 103621359 | 227786_AT_HG-U133B | chr8q24.11 | TRAP25 | 118499584 | 1.146115287 | 1.24E-04 | 1 | 0 |
| CNT_COMBINED_SL21 | 9 | 207964_X_AT_HG-U133A | chr9p21.3 | IFNA4 | 21176781 | 222618_AT_HG-U133B | chr9p21.1 | SMU-1 | 33034794 | -0.682010581 | 1 | 0.001076822 | 1.96E-05 |
| CNT_COMBINED_SL21 | 9 | 224412_S_AT_HG-U133B | chr9q21.13 | TRPM6 | 72794604 | 230645_AT_HG-U133B | chr9q21.32 | MGC20553 | 81319508 | -0.949299714 | 1 | 3.76E-04 | 7.66E-11 |
| CNT_COMBINED_SL21 | 9 | 226356_AT_HG-U133B | chr9q34.11 | FLJ14596 | 127208745 | 211979_AT_HG-U133A | chr9q34.11 | GPR107 | 128277938 | -0.662698425 | 1 | 0.004150391 | 0.007164052 |
| CNT_COMBINED_SL31 | 1 | 208783_S_AT_HG-U133A | chr1q32.2 | MCP | 205051907 | 217894_AT_HG-U133A | chr1q41 | KCTD3 | 212850982 | 0.890083626 | 4.67E-06 | 1 | 0 |
| CNT_COMBINED_SL31 | 1 | 211552_S_AT_HG-U133A | chr1p36.13 | ALDH4A1 | 18667448 | 201224_S_AT_HG-U133A | chr1p36.11 | SRRM1 | 24346894 | -1.2635067 | 1 | 5.92E-11 | 0 |
| CNT_COMBINED_SL31 | 10 | 205879_X_AT_HG-U133A | chr10q11.21 | RET | 42901440 | 212989_AT_HG-U133A | chr10q11.23 | MOB | 51410156 | -0.540997668 | 1 | 1.59E-05 | 3.47E-07 |
| CNT_COMBINED_SL31 | 10 | 238354_X_AT_HG-U133B | chr10p15.3 | Hs.432397 | 548637 | 200009_AT_HG-U133B | chr10p15.1 | GDI2 | 5811233 | -0.557893737 | 1 | 0.001045151 | 1.58E-05 |
| CNT_COMBINED_SL31 | 11 | 200766_AT_HG-U133A | chr11p15.5 | CTSD | 1738437 | 204804_AT_HG-U133A | chr11p15.4 | SSA1 | 4370507 | 0.73516129 | 1.15E-04 | 1 | 2.24E-10 |
| CNT_COMBINED_SL31 | 11 | 212832_S_AT_HG-U133A | chr11p11.2 | ch-TOG | 46729470 | 211240_X_AT_HG-U133A | chr11q12.1 | CTNND1 | 57360679 | 0.406177872 | 5.52E-04 | 1 | 0.003089904 |
| CNT_COMBINED_SL31 | 11 | 229072_AT_HG-U133B | chr11q14.1 | Hs.26339.0 | 82410510 | 214789_X_AT_HG-U133A | chr11q21 | SRP46 | 94489938 | 0.379341806 | 1.94E-04 | 1 | 0.004488841 |
| CNT_COMBINED_SL31 | 11 | 239848_AT_HG-U133B | chr11p13 | Hs.120054.0 | 32591666 | 213661_AT_HG-U133A | chr11p13 | DKFZP586H2123 | 35417713 | 0.441827954 | 9.34E-04 | 1 | 0.00771306 |
| CNT_COMBINED_SL31 | 11 | 244756_AT_HG-U133B | chr11q13.2 | LOC338692 | 66833601 | 201059_AT_HG-U133A | chr11q13.3 | EMS1 | 70008651 | -0.490841676 | 1 | 5.09E-06 | 3.70E-11 |
| CNT_COMBINED_SL31 | 12 | 200822_X_AT_HG-U133A | chr12p13.31 | TPI1 | 6849689 | 240572_S_AT_HG-U133B | chr12p13.31 | LOC196478 | 9697547 | -0.363440863 | 1 | 7.48E-04 | 4.89E-05 |
| CNT_COMBINED_SL31 | 12 | 234984_AT_HG-U133B | chr12q23.1 | NEDD1 | 95849361 | 220060_S_AT_HG-U133A | chr12q23.2 | FLJ20641 | 101092369 | 0.575197134 | 0.00126685 | 1 | 1.43E-04 |
| CNT_COMBINED_SL31 | 12 | 242273_AT_HG-U133B | chr12q14.3 | Hs.175569 | 64796618 | 235775_AT_HG-U133B | chr12q21.31 | DKFZp762A217 | 82030003 | 0.660266258 | 1.09E-09 | 1 | 0 |
| CNT_COMBINED_SL31 | 13 | 227490_AT_HG-U133B | chr13q14.3 | WDFY2 | 50128485 | 229097_AT_HG-U133B | chr13q21.2 | Hs.283127.0 | 58037761 | 2.908172059 | 0.009765625 | 1 | 0 |
| CNT_COMBINED_SL31 | 14 | 230418_S_AT_HG-U133B | chr14q24.1 | KIAA1130 | 67811080 | 203981_S_AT_HG-U133A | chr14q24.3 | ABCD4 | 72742907 | -0.648960572 | 1 | 4.13E-07 | 0 |
| CNT_COMBINED_SL31 | 15 | 201590_X_AT_HG-U133A | chr15q22.2 | ANXA2 | 58355630 | 223507_AT_HG-U133B | chr15q22.31 | CLPX | 63156476 | -0.715093591 | 1 | 9.18E-04 | 0.002361206 |
| CNT_COMBINED_SL31 | 15 | 211458_S_AT_HG-U133A | chr15q26.1 | GABARAPL3 | 88620616 | 203044_AT_HG-U133A | chr15q26.3 | CHSY1 | 99451245 | -0.986606216 | 1 | 1.76E-06 | 0 |
| CNT_COMBINED_SL31 | 15 | 228164_AT_HG-U133B | chr15q21.2 | AP4E1 | 49013579 | 221156_X_AT_HG-U133A | chr15q21.3 | CPR8 | 53367137 | -1.192789383 | 1 | 3.20E-04 | 0 |
| CNT_COMBINED_SL31 | 16 | 209616_S_AT_HG-U133A | chr16q12.2 | CES1 | 55612410 | 225770_AT_HG-U133B | chr16q13 | KIAA1972 | 57036697 | -1.164832385 | 1 | 3.98E-04 | 1.73E-07 |
| CNT_COMBINED_SL31 | 16 | 229223_AT_HG-U133B | chr16q22.1 | Hs.513684 | 68036147 | 220446_S_AT_HG-U133A | chr16q22.2 | CHST4 | 71348041 | 0.76430108 | 2.18E-06 | 1 | 0 |
| CNT_COMBINED_SL31 | 16 | 40149_AT_HG-U133A | chr16p11.2 | SH2B | 28922704 | 223179_AT_HG-U133B | chr16p11.2 | MGC10500 | 30141399 | -1.417866001 | 1 | 0.002197266 | 2.92E-09 |
| CNT_COMBINED_SL31 | 17 | 202192_S_AT_HG-U133A | chr17p13.1 | GAS7 | 10014521 | 239898_X_AT_HG-U133B | chr17p12 | ZNF286 | 15805077 | -0.560931896 | 1 | 3.07E-04 | 0.00656595 |
| CNT_COMBINED_SL31 | 17 | 221987_S_AT_HG-U133A | chr17p13.3 | FLJ10534 | 2433924 | 209003_AT_HG-U133A | chr17p13.2 | SLC25A11 | 5041349 | -0.965663081 | 1 | 6.06E-07 | 8.61E-14 |
| CNT_COMBINED_SL31 | 17 | 238694_AT_HG-U133B | chr17q23.2 | Hs.66692.0 | 55420308 | 202630_AT_HG-U133A | chr17q23.2 | APPBP2 | 58994985 | 1.078005867 | 3.62E-07 | 1 | 0 |
| CNT_COMBINED_SL31 | 18 | 212880_AT_HG-U133A | chr18q21.31 | WDR7 | 52845492 | 227542_AT_HG-U133B | chr18q22.2 | SOCS4 | 66145826 | -1.447414234 | 1 | 1.36E-05 | 0 |
| CNT_COMBINED_SL31 | 19 | 210463_X_AT_HG-U133A | chr19p13.2 | FLJ20244 | 13076747 | 203238_S_AT_HG-U133A | chr19p13.12 | NOTCH3 | 15131499 | 0.329354836 | 0.002380371 | 1 | 2.22E-08 |
| CNT_COMBINED_SL31 | 19 | 213523_AT_HG-U133A | chr19q12 | CCNE1 | 35006470 | 226700_AT_HG-U133B | chr19q13.12 | U2AF1L3 | 40925334 | 0.40823267 | 1.41E-06 | 1 | 0 |
| CNT_COMBINED_SL31 | 19 | 237372_AT_HG-U133B | chr19q13.31 | Hs.279807.0 | 48376703 | 202264_S_AT_HG-U133A | chr19q13.32 | TOMM40 | 50098207 | -0.42454143 | 1 | 4.31E-04 | 1.29E-05 |
| CNT_COMBINED_SL31 | 19 | 55705_AT_HG-U133A | chr19p13.3 | MGC16353 | 847546 | 227965_AT_HG-U133B | chr19p13.3 | APCL | 1408742 | -0.370918133 | 1 | 0.001235179 | 3.22E-04 |
| CNT_COMBINED_SL31 | 2 | 201595_S_AT_HG-U133A | chr2q32.1 | LEREPO4 | 187576068 | 225325_AT_HG-U133B | chr2q32.2 | FLJ20160 | 191568859 | 0.575186099 | 0.003601074 | 1 | 0.003201477 |
| CNT_COMBINED_SL31 | 2 | 205571_AT_HG-U133A | chr2q11.2 | LIPT1 | 99400416 | 206571_S_AT_HG-U133A | chr2q11.2 | MAP4K4 | 102126692 | -0.496112486 | 1 | 0.003601074 | 1.53E-07 |
| CNT_COMBINED_SL31 | 2 | 207979_S_AT_HG-U133A | chr2p11.2 | CD8B1 | 87043380 | 239624_AT_HG-U133B | chr2p11.2 | Hs.503451 | 91239193 | -0.712868305 | 1 | 0.001800537 | 3.60E-14 |
| CNT_COMBINED_SL31 | 2 | 213931_AT_HG-U133A | chr2p25.1 | Hs.502810 | 8844377 | 203797_AT_HG-U133A | chr2p24.2 | VSNL1 | 17821497 | 0.775302563 | 7.61E-07 | 1 | 6.09E-13 |
| CNT_COMBINED_SL31 | 2 | 225572_AT_HG-U133B | chr2q33.3 | CREB1 | 208670132 | 210495_X_AT_HG-U133A | chr2q35 | FN1 | 216428165 | 0.70996416 | 0.001868603 | 1 | 7.27E-06 |
| CNT_COMBINED_SL31 | 20 | 202878_S_AT_HG-U133A | chr20p11.21 | C1QR1 | 23055038 | 212312_AT_HG-U133A | chr20q11.21 | BCL2L1 | 30997306 | 1.398971402 | 0.004882813 | 1 | 0.004164639 |
| CNT_COMBINED_SL31 | 20 | 210040_AT_HG-U133A | chr20q13.12 | SLC12A5 | 45373634 | 203174_S_AT_HG-U133A | chr20q13.33 | ARFRP1 | 63057339 | 1.429852646 | 9.13E-10 | 1 | 0 |
| CNT_COMBINED_SL31 | 20 | 218089_AT_HG-U133A | chr20q11.23 | C20orf4 | 35559719 | 226973_AT_HG-U133B | chr20q11.23 | C20orf102 | 37258623 | 1.647311842 | 0.009765625 | 1 | 8.74E-08 |
| CNT_COMBINED_SL31 | 21 | 211626_X_AT_HG-U133A | chr21q22.2 | ERG | 38673944 | 243027_AT_HG-U133B | chr21q22.2 | LOC150084 | 40093956 | 0.565591398 | 0.004882813 | 1 | 4.97E-06 |
| CNT_COMBINED_SL31 | 22 | 226602_S_AT_HG-U133B | chr22q11.23 | BCR | 21984344 | 209919_X_AT_HG-U133A | chr22q11.23 | GGT1 | 23348420 | -1.399231953 | 1 | 6.10E-04 | 5.55E-15 |
| CNT_COMBINED_SL31 | 23 | 208598_S_AT_HG-U133A | chrXp11.22 | Hs.3383.1 | 52527078 | 243521_AT_HG-U133B | chrXp11.1 | Hs.224455 | 56898699 | 0.961032005 | 8.87E-04 | 1 | 3.68E-04 |
| CNT_COMBINED_SL31 | 23 | 226875_AT_HG-U133B | chrXq24 | FLJ32122 | 116571167 | 218668_S_AT_HG-U133A | chrXq26.2 | RAP2C | 130042718 | 1.338203658 | 4.62E-08 | 1 | 0 |
| CNT_COMBINED_SL31 | 3 | 201892_S_AT_HG-U133A | chr3p21.31 | IMPDH2 | 49020805 | 203246_S_AT_HG-U133A | chr3p21.31 | NPR2L | 50343974 | -0.395929343 | 1 | 1.22E-04 | 2.59E-06 |
| CNT_COMBINED_SL31 | 3 | 207808_S_AT_HG-U133A | chr3q11.2 | PROS1 | 94912918 | 201952_AT_HG-U133A | chr3q13.11 | ALCAM | 106616090 | 0.73134571 | 4.29E-05 | 1 | 4.12E-14 |
| CNT_COMBINED_SL31 | 3 | 211707_S_AT_HG-U133A | chr3q21.1 | KIAA0036 | 122809827 | 218703_AT_HG-U133A | chr3q21.1 | SEC22L2 | 124311661 | -0.428853047 | 1 | 0.006469727 | 4.63E-04 |
| CNT_COMBINED_SL31 | 3 | 231958_AT_HG-U133B | chr3p25.2 | DKFZp434E0519 | 11807093 | 202209_AT_HG-U133A | chr3p25.1 | LSM3 | 14195353 | 0.422734254 | 0.001617432 | 1 | 0.004179533 |
| CNT_COMBINED_SL31 | 4 | 207505_AT_HG-U133A | chr4q21.21 | PRKG2 | 82468358 | 212412_AT_HG-U133A | chr4q22.3 | LIM | 96047186 | -0.590177273 | 1 | 1.24E-04 | 2.44E-06 |
| CNT_COMBINED_SL31 | 4 | 226822_AT_HG-U133B | chr4q35.1 | Hs.21958.0 | 185639990 | 242822_AT_HG-U133B | chr4q35.2 | Hs.130535 | 191169554 | -0.883377393 | 1 | 2.09E-04 | 0 |
| CNT_COMBINED_SL31 | 4 | 228106_AT_HG-U133B | chr4p15.32 | FLJ20280 | 17553207 | 242959_AT_HG-U133B | chr4p14 | Hs.177264 | 37257780 | -0.89110459 | 1 | 2.59E-05 | 0 |
| CNT_COMBINED_SL31 | 5 | 218339_AT_HG-U133A | chr5q33.2 | MRPL22 | 154358967 | 209455_AT_HG-U133A | chr5q35.1 | FBXW1B | 171269635 | 0.445161287 | 2.48E-04 | 1 | 0.001775962 |
| CNT_COMBINED_SL31 | 5 | 224876_AT_HG-U133B | chr5q31.1 | FLJ37562 | 134271061 | 227404_S_AT_HG-U133B | chr5q31.2 | EGR1 | 137880750 | 0.694838712 | 8.73E-05 | 1 | 8.47E-09 |
| CNT_COMBINED_SL31 | 5 | 225326_AT_HG-U133B | chr5q32 | KIAA1311 | 145696645 | 213860_X_AT_HG-U133A | chr5q33.1 | CSNK1A1 | 148904253 | -0.648680356 | 1 | 0.009277344 | 1.53E-05 |
| CNT_COMBINED_SL31 | 5 | 231046_AT_HG-U133B | chr5q21.3 | Hs.158583.0 | 108139777 | 230311_S_AT_HG-U133B | chr5q23.2 | PRDM6 | 122599433 | -0.664587813 | 1 | 4.90E-07 | 0 |
| CNT_COMBINED_SL31 | 6 | 202727_S_AT_HG-U133A | chr6q23.3 | IFNGR1 | 137499344 | 237059_AT_HG-U133B | chr6q24.2 | Hs.12565 | 142525911 | 0.693548386 | 0.005615234 | 1 | 0.002650054 |
| CNT_COMBINED_SL31 | 6 | 202876_S_AT_HG-U133A | chr6p21.32 | PBX2 | 32224015 | 213540_AT_HG-U133A | chr6p21.32 | HSD17B8 | 33219978 | -0.659378734 | 1 | 0.001456877 | 0 |
| CNT_COMBINED_SL31 | 6 | 206074_S_AT_HG-U133A | chr6p21.31 | HMGA1 | 34260376 | 203160_S_AT_HG-U133A | chr6p21.2 | RNF8 | 37405378 | 0.468372268 | 0.002807617 | 1 | 0.002506017 |
| CNT_COMBINED_SL31 | 7 | 221911_AT_HG-U133A | chr7p21.2 | ETV1 | 13678217 | 209627_S_AT_HG-U133A | chr7p15.3 | OSBPL3 | 24578455 | 0.806953406 | 6.27E-05 | 1 | 0 |
| CNT_COMBINED_SL31 | 7 | 224681_AT_HG-U133B | chr7p22.3 | GNA12 | 2512650 | 210547_X_AT_HG-U133A | chr7p21.3 | ICA1 | 7897860 | 0.909438471 | 2.79E-06 | 1 | 0 |
| CNT_COMBINED_SL31 | 7 | 235301_AT_HG-U133B | chr7q21.12 | FLJ31340 | 86121421 | 226332_AT_HG-U133B | chr7q21.2 | Hs.470311 | 91802474 | -0.922365591 | 1 | 3.01E-04 | 0 |
| CNT_COMBINED_SL31 | 7 | 241986_AT_HG-U133B | chr7p14.3 | BMPER | 33936044 | 222429_AT_HG-U133B | chr7p13 | HIP-55 | 43841368 | 0.832895255 | 2.79E-06 | 1 | 0 |
| CNT_COMBINED_SL31 | 8 | 208323_S_AT_HG-U133A | chr8q24.13 | ANXA13 | 124649818 | 203760_S_AT_HG-U133A | chr8q24.22 | SLA | 134006556 | 1.06075268 | 8.89E-06 | 1 | 0 |
| CNT_COMBINED_SL31 | 8 | 226463_AT_HG-U133B | chr8q22.3 | ATP6V1C1 | 104041287 | 204501_AT_HG-U133A | chr8q24.12 | NOV | 120392282 | 1.002618038 | 1.49E-05 | 1 | 0 |
| CNT_COMBINED_SL31 | 9 | 231040_AT_HG-U133B | chr9q21.13 | Hs.184780.0 | 72764750 | 240547_AT_HG-U133B | chr9q21.32 | Hs.522256 | 81930051 | -0.730498531 | 1 | 2.59E-05 | 1.24E-14 |
| CNT_COMBINED_SL41 | 1 | 220688_S_AT_HG-U133A | chr1p36.13 | C1orf33 | 19053453 | 202553_S_AT_HG-U133A | chr1p36.11 | P29 | 24900186 | -1.185652794 | 1 | 2.14E-11 | 0 |
| CNT_COMBINED_SL41 | 1 | 229659_S_AT_HG-U133B | chr1q32.1 | PIGR | 204185613 | 220435_AT_HG-U133A | chr1q41 | DKFZp547M236 | 217143939 | 0.690609755 | 9.78E-09 | 1 | 0 |
| CNT_COMBINED_SL41 | 10 | 243054_AT_HG-U133B | chr10p15.3 | Hs.255096 | 142962 | 222824_AT_HG-U133B | chr10p13 | SEC61A2 | 12211420 | -0.414965258 | 1 | 4.64E-07 | 0 |
| CNT_COMBINED_SL41 | 11 | 208714_AT_HG-U133A | chr11q13.2 | NDUFV1 | 67154318 | 200747_S_AT_HG-U133A | chr11q13.4 | NUMA1 | 71440259 | -0.417987804 | 1 | 7.23E-05 | 6.28E-11 |
| CNT_COMBINED_SL41 | 11 | 234525_AT_HG-U133B | chr11q14.1 | Hs.285786.0 | 83825339 | 203212_S_AT_HG-U133A | chr11q21 | MTMR2 | 95254286 | 0.405949742 | 1.92E-06 | 1 | 4.74E-05 |
| CNT_COMBINED_SL41 | 12 | 238651_AT_HG-U133B | chr12q15 | Hs.23096 | 67921640 | 235775_AT_HG-U133B | chr12q21.31 | DKFZp762A217 | 82030003 | 0.680055074 | 3.78E-07 | 1 | 0 |
| CNT_COMBINED_SL41 | 13 | 216870_X_AT_HG-U133A | chr13q14.2 | DLEU2 | 48415744 | 225578_AT_HG-U133B | chr13q22.1 | Hs.28465.1 | 71080528 | 2.447096382 | 2.59E-05 | 1 | 0 |
| CNT_COMBINED_SL41 | 14 | 224445_S_AT_HG-U133B | chr14q32.33 | ZFYVE21 | 102189637 | 32137_AT_HG-U133A | chr14q32.33 | JAG2 | 103579806 | -0.694208049 | 1 | 0.00177002 | 0 |
| CNT_COMBINED_SL41 | 14 | 239600_AT_HG-U133B | chr14q24.2 | Hs.191850.0 | 69922896 | 203981_S_AT_HG-U133A | chr14q24.3 | ABCD4 | 72742907 | -0.605523677 | 1 | 3.20E-04 | 1.61E-14 |
| CNT_COMBINED_SL41 | 15 | 205510_S_AT_HG-U133A | chr15q21.2 | GABPB2 | 48361919 | 222630_AT_HG-U133B | chr15q21.3 | FLJ12994 | 54098860 | -0.953353666 | 1 | 1.40E-05 | 0 |
| CNT_COMBINED_SL41 | 15 | 223468_S_AT_HG-U133B | chr15q26.1 | RGMA | 91316462 | 225035_X_AT_HG-U133B | chr15q26.3 | FLJ25222 | 100251393 | -1.103602918 | 1 | 1.12E-04 | 0 |
| CNT_COMBINED_SL41 | 16 | 208932_AT_HG-U133A | chr16p11.2 | PPP4C | 30133657 | 202525_AT_HG-U133A | chr16p11.2 | PRSS8 | 31178962 | -0.669910814 | 1 | 6.17E-04 | 9.96E-04 |
| CNT_COMBINED_SL41 | 16 | 216623_X_AT_HG-U133A | chr16q12.1 | TNRC9 | 52250601 | 208581_X_AT_HG-U133A | chr16q12.2 | MT1X | 56491802 | -0.856167549 | 1 | 4.50E-05 | 1.15E-05 |
| CNT_COMBINED_SL41 | 16 | 232057_AT_HG-U133B | chr16q22.1 | FLJ13291 | 68110823 | 200687_S_AT_HG-U133A | chr16q22.1 | SF3B3 | 70381781 | 0.647786812 | 2.00E-06 | 1 | 0 |
| CNT_COMBINED_SL41 | 17 | 204485_S_AT_HG-U133A | chr17q22 | TOM1L1 | 53513482 | 204566_AT_HG-U133A | chr17q23.2 | PPM1D | 59215716 | 0.91085366 | 3.27E-09 | 1 | 0 |
| CNT_COMBINED_SL41 | 17 | 215354_S_AT_HG-U133A | chr17p13.2 | PELP1 | 4781276 | 219123_AT_HG-U133A | chr17p13.2 | ZNF232 | 5209632 | -0.777392121 | 1 | 0.002624512 | 1.50E-04 |
| CNT_COMBINED_SL41 | 18 | 218247_S_AT_HG-U133A | chr18q21.1 | LOC51320 | 46952989 | 233228_AT_HG-U133B | chr18q22.3 | Hs.283338.0 | 70626973 | -1.250321231 | 1 | 1.08E-07 | 0 |
| CNT_COMBINED_SL41 | 19 | 225254_AT_HG-U133B | chr19q13.2 | MGC20255 | 46521454 | 227327_AT_HG-U133B | chr19q13.2 | EGFL4 | 47574314 | -0.366666665 | 1 | 0.00126685 | 0.001025154 |
| CNT_COMBINED_SL41 | 19 | 226786_AT_HG-U133B | chr19p13.13 | RFX1 | 13933608 | 44702_AT_HG-U133A | chr19p13.12 | 4877366_RC | 15086197 | 0.316869915 | 0.009765625 | 1 | 1.34E-05 |
| CNT_COMBINED_SL41 | 19 | 242919_AT_HG-U133B | chr19p13.11 | LOC114977 | 19865189 | 204122_AT_HG-U133A | chr19q13.12 | TYROBP | 41087287 | 0.306752011 | 8.64E-07 | 1 | 0 |
| CNT_COMBINED_SL41 | 2 | 200705_S_AT_HG-U133A | chr2q33.3 | EEF1B2 | 207227918 | 219648_AT_HG-U133A | chr2q35 | FLJ10116 | 217010116 | 0.544085376 | 3.40E-04 | 1 | 0.003973643 |
| CNT_COMBINED_SL41 | 2 | 219873_AT_HG-U133A | chr2p25.3 | COLEC11 | 3256001 | 209683_AT_HG-U133A | chr2p24.2 | DKFZP566A1524 | 16715980 | 0.700800896 | 8.57E-08 | 1 | 0 |
| CNT_COMBINED_SL41 | 2 | 221575_AT_HG-U133A | chr2q37.3 | SCLY | 239294237 | 216945_X_AT_HG-U133A | chr2q37.3 | PASK | 242366371 | -0.391269585 | 1 | 0.006347656 | 3.48E-08 |
| CNT_COMBINED_SL41 | 2 | 222704_AT_HG-U133B | chr2p11.2 | LOC90784 | 86228055 | 239624_AT_HG-U133B | chr2p11.2 | Hs.503451 | 91239193 | -0.409294881 | 1 | 9.72E-04 | 1.61E-12 |
| CNT_COMBINED_SL41 | 20 | 212947_AT_HG-U133A | chr20q13.13 | SLC9A8 | 49193607 | 204378_AT_HG-U133A | chr20q13.2 | BCAS1 | 53245993 | 1.818118462 | 7.63E-04 | 1 | 0 |
| CNT_COMBINED_SL41 | 20 | 89476_R_AT_HG-U133A | chr20q13.32 | NPEPL1 | 57973951 | 216088_S_AT_HG-U133A | chr20q13.33 | PSMA7 | 61399634 | 1.653252025 | 0.003051758 | 1 | 7.36E-11 |
| CNT_COMBINED_SL41 | 21 | 241726_AT_HG-U133B | chr21q22.13 | HLCS | 37043253 | 202086_AT_HG-U133A | chr21q22.3 | MX1 | 41750953 | 0.333042976 | 0.002685547 | 1 | 6.80E-08 |
| CNT_COMBINED_SL41 | 22 | 234764_X_AT_HG-U133B | chr22q11.22 | Hs.307341.0 | 21036919 | 54037_AT_HG-U133A | chr22q12.1 | HPS4 | 25172642 | -1.039170728 | 1 | 3.24E-06 | 0 |
| CNT_COMBINED_SL41 | 23 | 200657_AT_HG-U133A | chrXq24 | SLC25A5 | 117358182 | 230113_AT_HG-U133B | chrXq26.2 | Hs.105618 | 130208999 | 1.230284558 | 3.61E-07 | 1 | 0 |
| CNT_COMBINED_SL41 | 3 | 207808_S_AT_HG-U133A | chr3q11.2 | PROS1 | 94912918 | 227181_AT_HG-U133B | chr3q12.2 | LOC348801 | 101495624 | 0.769764135 | 0.002624512 | 1 | 1.24E-13 |
| CNT_COMBINED_SL41 | 3 | 209177_AT_HG-U133A | chr3p21.31 | DKFZP564J0123 | 49018813 | 205417_S_AT_HG-U133A | chr3p21.31 | DAG1 | 49531502 | -0.399155722 | 1 | 0.002624512 | 1.48E-04 |
| CNT_COMBINED_SL41 | 3 | 227503_AT_HG-U133B | chr3q13.13 | Hs.23762.0 | 112240615 | 200776_S_AT_HG-U133A | chr3q13.31 | Hs.447695 | 117685318 | -0.370426827 | 1 | 2.07E-05 | 1.32E-07 |
| CNT_COMBINED_SL41 | 4 | 222738_AT_HG-U133B | chr4q35.1 | BOMB | 184935302 | 226745_AT_HG-U133B | chr4q35.2 | Hs.237642.0 | 187829479 | -0.749227622 | 1 | 8.73E-05 | 5.06E-14 |
| CNT_COMBINED_SL41 | 4 | 228992_AT_HG-U133B | chr4p15.32 | EG1 | 17377769 | 226139_AT_HG-U133B | chr4p15.2 | DKFZp761B107 | 24558644 | -0.870121943 | 1 | 0.001159668 | 0 |
| CNT_COMBINED_SL41 | 5 | 202396_AT_HG-U133A | chr5q32 | TCERG1 | 145919038 | 227717_AT_HG-U133B | chr5q33.1 | Hs.144871.0 | 149042586 | -0.511280489 | 1 | 0.004638672 | 2.25E-04 |
| CNT_COMBINED_SL41 | 5 | 230725_AT_HG-U133B | chr5q15 | Hs.444378 | 94111725 | 222637_AT_HG-U133B | chr5q23.1 | PTD002 | 115704655 | -0.563244468 | 1 | 8.05E-09 | 0 |
| CNT_COMBINED_SL41 | 6 | 212554_AT_HG-U133A | chr6p22.3 | CAP2 | 17665419 | 207051_AT_HG-U133A | chr6p22.2 | SLC17A4 | 25887865 | -0.469756094 | 1 | 0.001456877 | 0 |
| CNT_COMBINED_SL41 | 6 | 237619_AT_HG-U133B | chr6p25.2 | C6orf146 | 4013638 | 223105_S_AT_HG-U133B | chr6p24.2 | C6orf53 | 10838880 | 0.704166671 | 1.32E-05 | 1 | 0 |
| CNT_COMBINED_SL41 | 7 | 209993_AT_HG-U133A | chr7q21.12 | ABCB1 | 86745402 | 204873_AT_HG-U133A | chr7q21.2 | PEX1 | 91728398 | -0.912764227 | 1 | 0.001171836 | 0 |
| CNT_COMBINED_SL41 | 7 | 212447_AT_HG-U133A | chr7p14.3 | BKLHD1 | 32650046 | 220106_AT_HG-U133A | chr7p13 | NPC1L1 | 44292980 | 0.763150953 | 1.02E-07 | 1 | 0 |
| CNT_COMBINED_SL41 | 7 | 227129_X_AT_HG-U133B | chr7q32.1 | Hs.326048.2 | 127848431 | 239331_AT_HG-U133B | chr7q32.3 | Hs.184721 | 130005831 | 0.748102983 | 0.001171836 | 1 | 1.93E-04 |
| CNT_COMBINED_SL41 | 8 | 202393_S_AT_HG-U133A | chr8q22.3 | TIEG | 103617650 | 202241_AT_HG-U133A | chr8q24.13 | C8FW | 126406751 | 0.936359524 | 1.87E-12 | 1 | 0 |
| CNT_COMBINED_SL41 | 8 | 202626_S_AT_HG-U133A | chr8q12.1 | LYN | 56972640 | 217590_S_AT_HG-U133A | chr8q21.11 | ANKTM1 | 72984467 | 0.758708243 | 1.88E-06 | 1 | 0 |
| CNT_COMBINED_SL41 | 9 | 224209_S_AT_HG-U133B | chr9q21.12 | GDA | 70322786 | 203221_AT_HG-U133A | chr9q21.32 | TLE1 | 79655815 | -0.611032969 | 1 | 9.19E-05 | 2.28E-12 |
| CNT_COMBINED_SL51 | 1 | 220962_S_AT_HG-U133A | chr1p36.13 | PADI1 | 16951873 | 227818_AT_HG-U133B | chr1p36.11 | DKFZP434L0117 | 26209447 | -1.088651577 | 1 | 1.87E-14 | 0 |
| CNT_COMBINED_SL51 | 1 | 223851_S_AT_HG-U133B | chr1p36.33 | TNFRSF18 | 1045291 | 208009_S_AT_HG-U133A | chr1p36.32 | ARHGEF16 | 3180072 | -0.920475907 | 1 | 5.10E-06 | 4.52E-13 |
| CNT_COMBINED_SL51 | 1 | 228528_AT_HG-U133B | chr1q32.2 | Hs.290825.0 | 205058576 | 228121_AT_HG-U133B | chr1q41 | TGFB2 | 215673320 | 0.665019606 | 1.08E-06 | 1 | 0 |
| CNT_COMBINED_SL51 | 10 | 222824_AT_HG-U133B | chr10p13 | SEC61A2 | 12211420 | 229713_AT_HG-U133B | chr10p12.2 | Hs.57079.0 | 22829186 | -0.256697627 | 1 | 1.32E-04 | 4.75E-11 |
| CNT_COMBINED_SL51 | 10 | 236310_AT_HG-U133B | chr10q11.21 | Hs.173866.0 | 42297074 | 239151_AT_HG-U133B | chr10q11.22 | Hs.314437.2 | 46594256 | -0.47286326 | 1 | 0.008300781 | 8.21E-05 |
| CNT_COMBINED_SL51 | 11 | 222759_AT_HG-U133B | chr11q13.2 | CGI-85 | 67708616 | 221845_S_AT_HG-U133A | chr11q13.4 | SKD3 | 71730671 | -0.343631711 | 1 | 0.001678576 | 1.70E-07 |
| CNT_COMBINED_SL51 | 11 | 228094_AT_HG-U133B | chr11q23.3 | AMICA | 117602373 | 200825_S_AT_HG-U133A | chr11q23.3 | HYOU1 | 118452581 | -0.379128536 | 1 | 0.001678576 | 1.22E-08 |
| CNT_COMBINED_SL51 | 12 | 203999_AT_HG-U133A | chr12q21.2 | SYT1 | 78347779 | 225885_AT_HG-U133B | chr12q22 | Hs.403150 | 91668803 | 0.489590019 | 3.08E-05 | 1 | 3.62E-07 |
| CNT_COMBINED_SL51 | 12 | 213761_AT_HG-U133A | chr12q15 | MDM1 | 66974693 | 219937_AT_HG-U133A | chr12q21.1 | TRHDE | 71345170 | 0.695607844 | 8.73E-05 | 1 | 8.44E-15 |
| CNT_COMBINED_SL51 | 12 | 224048_AT_HG-U133B | chr12q22 | USP44 | 94414052 | 223798_AT_HG-U133B | chr12q23.3 | DKFZP434K0427 | 103701268 | 0.362377454 | 8.21E-07 | 1 | 2.69E-06 |
| CNT_COMBINED_SL51 | 12 | 225528_AT_HG-U133B | chr12p11.21 | IPO8 | 30673246 | 227948_AT_HG-U133B | chr12p11.21 | FRABIN | 32688632 | -0.278332819 | 1 | 0.009277344 | 0.009966748 |
| CNT_COMBINED_SL51 | 13 | 226782_AT_HG-U133B | chr13q14.13 | LOC253512 | 43765485 | 204624_AT_HG-U133A | chr13q14.3 | ATP7B | 50305017 | 2.300366871 | 1.62E-07 | 1 | 0 |
| CNT_COMBINED_SL51 | 14 | 213927_AT_HG-U133A | chr14q24.2 | Hs.170267.0 | 69179310 | 225980_AT_HG-U133B | chr14q24.3 | C14orf117 | 72171896 | -0.59681046 | 1 | 9.18E-04 | 0 |
| CNT_COMBINED_SL51 | 14 | 226325_AT_HG-U133B | chr14q32.33 | ADSSL1 | 103182865 | 234882_AT_HG-U133B | chr14q32.33 | Hs.248015.0 | 104137870 | -0.699929416 | 1 | 0.003540039 | 0 |
| CNT_COMBINED_SL51 | 15 | 202766_S_AT_HG-U133A | chr15q21.1 | FBN1 | 46418376 | 207121_S_AT_HG-U133A | chr15q21.2 | MAPK6 | 50074015 | -0.7751634 | 1 | 9.50E-04 | 1.01E-04 |
| CNT_COMBINED_SL51 | 15 | 218415_AT_HG-U133A | chr15q26.1 | VPS33B | 89271710 | 225035_X_AT_HG-U133B | chr15q26.3 | FLJ25222 | 100251393 | -0.938921285 | 1 | 5.12E-06 | 0 |
| CNT_COMBINED_SL51 | 15 | 243198_AT_HG-U133B | chr15q21.3 | Hs.373484 | 54420582 | 226354_AT_HG-U133B | chr15q22.2 | LACTB | 61149495 | -0.813019621 | 1 | 1.96E-04 | 3.31E-13 |
| CNT_COMBINED_SL51 | 16 | 202659_AT_HG-U133A | chr16q22.1 | PSMB10 | 67744699 | 213407_AT_HG-U133A | chr16q22.2 | KIAA0931 | 71455194 | 0.475803925 | 9.37E-08 | 1 | 0 |
| CNT_COMBINED_SL51 | 16 | 229043_AT_HG-U133B | chr16q12.1 | TRF4-2 | 50043326 | 212859_X_AT_HG-U133A | chr16q12.2 | MT2A | 56434922 | -0.658701628 | 1 | 4.64E-05 | 5.53E-06 |
| CNT_COMBINED_SL51 | 16 | 243124_AT_HG-U133B | chr16p11.2 | Hs.499011 | 29165364 | 212046_X_AT_HG-U133A | chr16p11.2 | MAPK3 | 30162793 | -0.971907318 | 1 | 0.008300781 | 3.87E-06 |
| CNT_COMBINED_SL51 | 17 | 201528_AT_HG-U133A | chr17p13.3 | RPA1 | 2007531 | 214246_X_AT_HG-U133A | chr17p13.2 | CHRNE | 5001813 | -0.766020744 | 1 | 1.38E-07 | 3.27E-12 |
| CNT_COMBINED_SL51 | 17 | 209849_S_AT_HG-U133A | chr17q23.2 | RAD51C | 57255278 | 218991_AT_HG-U133A | chr17q23.2 | FLJ22087 | 58595062 | 0.768235296 | 0.001525879 | 1 | 2.77E-09 |
| CNT_COMBINED_SL51 | 17 | 223221_AT_HG-U133B | chr17p13.1 | SCO1 | 10784705 | 202078_AT_HG-U133A | chr17p11.2 | COPS3 | 17350831 | -0.397915966 | 1 | 0.003051758 | 0.004406487 |
| CNT_COMBINED_SL51 | 17 | 225339_AT_HG-U133B | chr17q21.33 | SPAG9 | 49514213 | 218630_AT_HG-U133A | chr17q23.2 | FLJ20345 | 56757498 | 0.77239963 | 6.82E-05 | 1 | 2.22E-14 |
| CNT_COMBINED_SL51 | 18 | 48580_AT_HG-U133A | chr18q21.1 | CGBP | 46060772 | 225407_AT_HG-U133B | chr18q23 | MBP | 72851804 | -1.13713904 | 1 | 4.82E-06 | 0 |
| CNT_COMBINED_SL51 | 19 | 207205_AT_HG-U133A | chr19q13.2 | CEACAM4 | 46817223 | 203655_AT_HG-U133A | chr19q13.31 | XRCC1 | 48739375 | -0.319607841 | 1 | 4.42E-04 | 6.59E-05 |
| CNT_COMBINED_SL51 | 19 | 238791_AT_HG-U133B | chr19p13.11 | LOC163227 | 21698308 | 202475_AT_HG-U133A | chr19q13.12 | NIFIE14 | 40729301 | 0.357474537 | 2.43E-06 | 1 | 0 |
| CNT_COMBINED_SL51 | 2 | 200768_S_AT_HG-U133A | chr2p11.2 | MAT2A | 85746405 | 224971_AT_HG-U133B | chr2q11.2 | MRPL30 | 99434712 | -0.256401133 | 1 | 3.15E-06 | 0 |
| CNT_COMBINED_SL51 | 2 | 218497_S_AT_HG-U133A | chr2p25.3 | RNASEH1 | 3157723 | 208638_AT_HG-U133A | chr2p25.1 | ATP6V1C2 | 10945351 | 0.734919748 | 8.88E-06 | 1 | 0 |
| CNT_COMBINED_SL51 | 2 | 226446_AT_HG-U133B | chr2q37.3 | HES6 | 239433707 | 204511_AT_HG-U133A | chr2q37.3 | FARP2 | 242754412 | -0.431957091 | 1 | 3.40E-04 | 0 |
| CNT_COMBINED_SL51 | 2 | 235078_AT_HG-U133B | chr2q33.2 | Hs.163603 | 204291888 | 225040_S_AT_HG-U133B | chr2q34 | RPE | 211088492 | 0.462992778 | 2.01E-04 | 1 | 3.73E-04 |
| CNT_COMBINED_SL51 | 20 | 216438_S_AT_HG-U133A | chr20q13.13 | Hs.288031.2 | 50142257 | 32723_AT_HG-U133A | chr20q13.31 | CSTF1 | 55664300 | 1.69604826 | 0.001708984 | 1 | 0 |
| CNT_COMBINED_SL51 | 21 | 211065_X_AT_HG-U133A | chr21q22.3 | PFKL | 44602915 | 213428_S_AT_HG-U133A | chr21q22.3 | COL6A1 | 46280725 | -0.327588797 | 1 | 0.005859375 | 0.001175219 |
| CNT_COMBINED_SL51 | 21 | 213989_X_AT_HG-U133A | chr21q22.12 | C21orf18 | 36336480 | 202086_AT_HG-U133A | chr21q22.3 | MX1 | 41750953 | 0.255746607 | 3.66E-04 | 1 | 1.14E-07 |
| CNT_COMBINED_SL51 | 22 | 218681_S_AT_HG-U133A | chr22q11.21 | SDF2L1 | 20321722 | 243816_AT_HG-U133B | chr22q11.23 | Hs.173012.0 | 22405485 | -0.935163399 | 1 | 0.002197266 | 3.00E-15 |
| CNT_COMBINED_SL51 | 22 | 223039_AT_HG-U133B | chr22q11.23 | MGC1842 | 23261048 | 204079_AT_HG-U133A | chr22q12.1 | TPST2 | 25246332 | -0.758496736 | 1 | 0.002197266 | 6.06E-04 |
| CNT_COMBINED_SL51 | 23 | 213666_AT_HG-U133A | chrXq24 | Sep 06 | 117503821 | 201828_X_AT_HG-U133A | chrXq26.3 | CXX1 | 132872615 | 1.053877996 | 1.65E-08 | 1 | 0 |
| CNT_COMBINED_SL51 | 3 | 207808_S_AT_HG-U133A | chr3q11.2 | PROS1 | 94912918 | 214143_X_AT_HG-U133A | chr3q12.3 | RPL24 | 102720867 | 0.440209433 | 0.001393535 | 1 | 4.09E-09 |
| CNT_COMBINED_SL51 | 3 | 212646_AT_HG-U133A | chr3p25.1 | RAFTLIN | 16332419 | 207044_AT_HG-U133A | chr3p24.2 | THRB | 24139416 | 0.317908501 | 0.005371094 | 1 | 0.001601881 |
| CNT_COMBINED_SL51 | 3 | 219734_AT_HG-U133A | chr3q13.2 | FLJ20174 | 114668718 | 204971_AT_HG-U133A | chr3q21.1 | CSTA | 123365034 | -0.366761328 | 1 | 9.51E-07 | 1.64E-13 |
| CNT_COMBINED_SL51 | 3 | 40020_AT_HG-U133A | chr3p21.31 | CELSR3 | 48634519 | 200059_S_AT_HG-U133B | chr3p21.31 | ARHA | 49356004 | -0.284923744 | 1 | 0.001393535 | 1.61E-05 |
| CNT_COMBINED_SL51 | 4 | 203914_X_AT_HG-U133A | chr4q34.1 | HPGD | 176107420 | 227337_AT_HG-U133B | chr4q35.1 | Lrp2bp | 187014310 | -0.743922893 | 1 | 6.73E-04 | 2.34E-13 |
| CNT_COMBINED_SL51 | 4 | 225014_AT_HG-U133B | chr4p15.2 | Hs.235026.0 | 25681729 | 240704_AT_HG-U133B | chr4p14 | Hs.118993 | 38883392 | -0.80509804 | 1 | 0.001098633 | 0 |
| CNT_COMBINED_SL51 | 4 | 228106_AT_HG-U133B | chr4p15.32 | FLJ20280 | 17553207 | 240095_AT_HG-U133B | chr4p15.2 | Hs.129636 | 24556082 | -0.790659541 | 1 | 0.008789063 | 2.10E-09 |
| CNT_COMBINED_SL51 | 5 | 222423_AT_HG-U133B | chr5q31.3 | NDFIP1 | 141561947 | 208645_S_AT_HG-U133A | chr5q33.1 | Hs.497875 | 149854965 | -0.376274512 | 1 | 4.77E-05 | 1.79E-09 |
| CNT_COMBINED_SL51 | 5 | 223449_AT_HG-U133B | chr5q23.1 | SEMA6A | 115855577 | 231171_AT_HG-U133B | chr5q23.2 | Hs.97104 | 127426968 | -0.396532509 | 1 | 6.54E-04 | 2.86E-06 |
| CNT_COMBINED_SL51 | 5 | 225176_AT_HG-U133B | chr5q15 | Hs.356079 | 96446730 | 219736_AT_HG-U133A | chr5q22.3 | TRIM36 | 114536807 | -0.585620914 | 1 | 2.12E-06 | 0 |
| CNT_COMBINED_SL51 | 6 | 203814_S_AT_HG-U133A | chr6p25.2 | NQO2 | 2957812 | 224450_S_AT_HG-U133B | chr6p24.3 | RIOK1 | 7362687 | 0.711987693 | 3.81E-05 | 1 | 0 |
| CNT_COMBINED_SL51 | 7 | 211747_S_AT_HG-U133A | chr7p14.3 | LSM5 | 32268968 | 224948_AT_HG-U133B | chr7p13 | MRPS24 | 43646958 | 0.757464055 | 5.29E-07 | 1 | 0 |
| CNT_COMBINED_SL51 | 7 | 219332_AT_HG-U133A | chr7p22.3 | FLJ23471 | 1218355 | 224452_S_AT_HG-U133B | chr7p22.1 | MGC12966 | 6113445 | 0.687247763 | 3.32E-05 | 1 | 1.38E-08 |
| CNT_COMBINED_SL51 | 8 | 205609_AT_HG-U133A | chr8q23.1 | ANGPT1 | 108218335 | 224650_AT_HG-U133B | chr8q24.12 | MAL2 | 120213983 | 0.850703569 | 3.20E-04 | 1 | 7.36E-05 |
| CNT_COMBINED_SL51 | 8 | 221504_S_AT_HG-U133A | chr8q11.23 | ATP6V1H | 54678345 | 221749_AT_HG-U133A | chr8q12.3 | FLJ31657 | 64174781 | 0.685129057 | 3.20E-04 | 1 | 3.82E-04 |
| CNT_COMBINED_SL51 | 8 | 226294_X_AT_HG-U133B | chr8q24.13 | FLJ23790 | 124783691 | 212149_AT_HG-U133A | chr8q24.22 | KIAA0143 | 132982022 | 0.792897605 | 1.90E-04 | 1 | 5.87E-08 |
| CNT_COMBINED_SL51 | 9 | 202387_AT_HG-U133A | chr9p13.3 | BAG1 | 33245093 | 201692_AT_HG-U133A | chr9p13.3 | OPRS1 | 34624840 | -0.345790081 | 1 | 3.87E-04 | 0.00702895 |
| CNT_COMBINED_SL51 | 9 | 235484_AT_HG-U133B | chr9q21.11 | Hs.173519.0 | 67782835 | 227865_AT_HG-U133B | chr9q21.32 | Hs.103158.0 | 81715585 | -0.498517569 | 1 | 1.27E-06 | 1.17E-10 |
